# Supplementary material for: Copper-Catalyzed Diboron-Mediated cis-Semi-Hydrogenation of Alkynes under Facile Conditions
Source: Molecules. 2022 Oct 25;27(21):7213. doi: 10.3390/molecules27217213 (PMC9656742; doi:10.3390/molecules27217213)

Supplementary Materials

Copper-catalyzed diboron-mediated *cis*-semihydrogenation of alkynes under facile conditions

Yuxi Zeng, Honggang Zhang, Daofan Ma*, Guangwei Wang*

Department of Chemistry, School of Science, Tianjin University, Tianjin 300354, China

*Correspondence: daofanma@163.com; wanggw@tju.edu.cn

Table of Contents

1. Experimental procedures of mechanistic study……………………………………S2

2. Possible explanation for reduced stereoselectivity in **2c**, **2d**, **2f**, and **2q** cases ………………………………………………………………………………S3

3. ^{1}H and ^{13}C NMR spectra for all compounds………………………………………S4

1. Experimental procedures of mechanistic studies

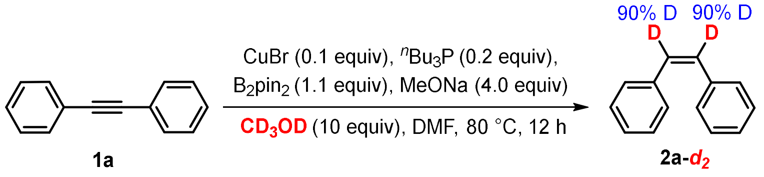

Synthesis of (*Z*)-1,2-diphenylethene-1,2-*d*$_2$ (2a-*d*$_2$). To a mixture of **1a** (71.2 mg, 0.4 mmol, 1.0 equiv), CuBr (5.8 mg, 0.04 mmol, 0.1 equiv), ^{n}Bu$_3$P (20 μL, 0.08 mmol, 0.2 equiv), B_2pin_2 (111.7 mg, 0.44 mmol, 1.1 equiv), NaOMe (86.4 mg, 1.6 mmol, 4 equiv) and CD_3OD (0.16 mL, 4 mmol, 10 equiv) was added 4.0 mL of DMF under argon. The reaction mixture was then placed in a preheated oil bath at 80 °C for 12 h. After the reaction was completed, the reaction was diluted with 8 mL of water then extracted with ethyl acetate (3 x 8 mL). The combined organic extracts were dried over anhydrous $MgSO_4$, filtered, and then concentrated under reduced pressure and the residue was further purified by silica gel column chromatography (PE) to give the target product **2a-*d*$_2$** (60.5 mg, 83% yield, 90% D by ^{1}H NMR). ^{1}H NMR (400 MHz, $CDCl_3$) δ 7.25 – 7.10 (m, 10H), 6.52 (s, 0.2H)

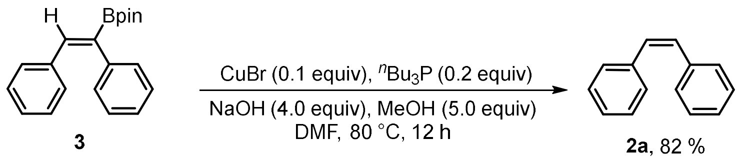

Alcoholysis of (*Z*)-2-(1,2-diphenylvinyl)-4,4,5,5-tetramethyl-1,3,2-dioxaborolane (3). To a mixture of **3** (63.6 mg, 0.2 mmol, 1.0 equiv), CuBr (2.9 mg, 0.02 mmol, 0.1 equiv), ^{n}Bu$_3$P (10 μL, 0.04 mmol, 0.2 equiv), NaOH (32.0 mg, 0.8 mmol, 4.0 equiv) and MeOH (40 μL, 2.0 mmol, 5.0 equiv) was added 4.0 mL of DMF under argon. The reaction mixture was then placed in a preheated oil bath at 80 °C for 12 h. After the reaction was completed, the reaction was monitored by GC analysis.

2. Possible explanation for reduced stereoselectivity in 2c, 2d, 2f, and 2q cases.

The reduced stereoselectivity of **2c** and **2d** might be due to the presence of electron donating group at *para*-position of arenes. These electron donating group might promote the isomerization through a reversible addition-elimination process as shown below. In the presence of electron donating group, the *in situ* generated olefin would be more labile to complex with another molecule of copper-boron species. Then addition of copper-boron species on double bond would lead to a saturated product, which could afford an isomerization product after elimination reaction. For the reduced stereoselectivity of **2f** and **2g**, we still have no convincing explanation. Actually, the stereoselectivity is the result of combination effects of steric hindrance and electronic effects of functional groups, making it difficult to predict which factor predominates.

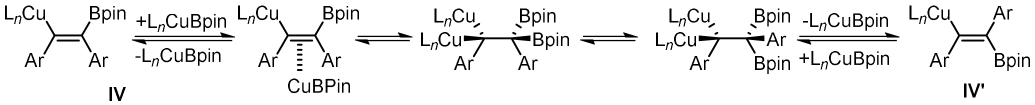

IV **IV'**

3. ^{1}H and ^{13}C NMR spectra for all compounds

(a) ^{1}H NMR, ^{13}C NMR, ^{19}F NMR and HRMS spectra of 2a-2w.

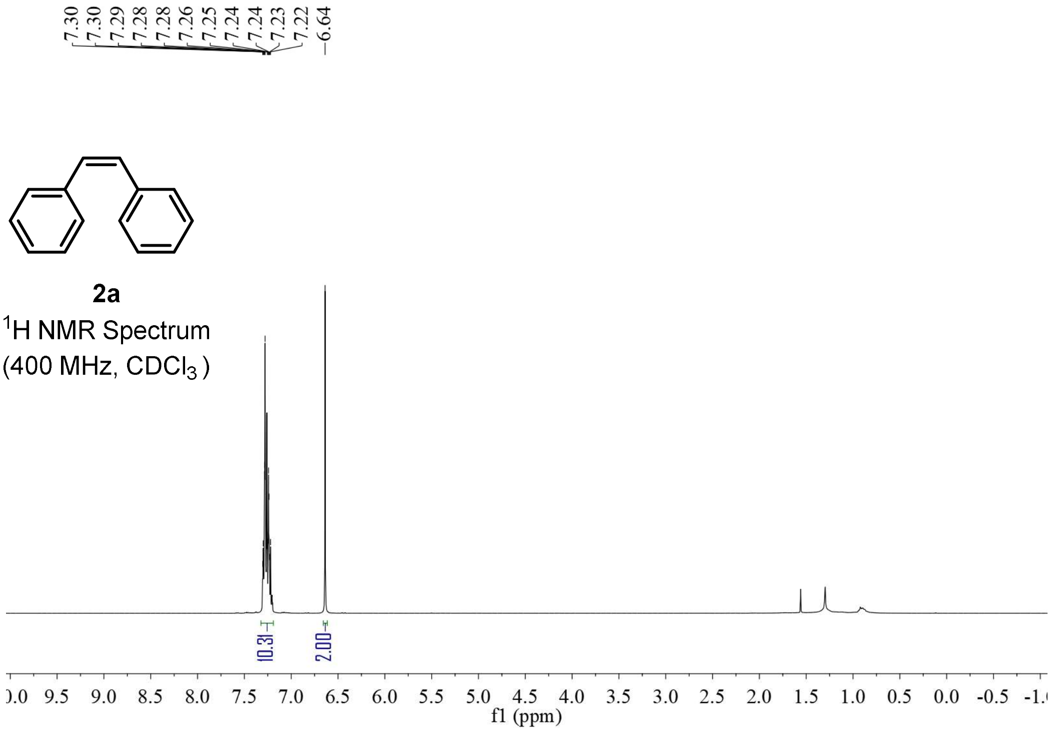

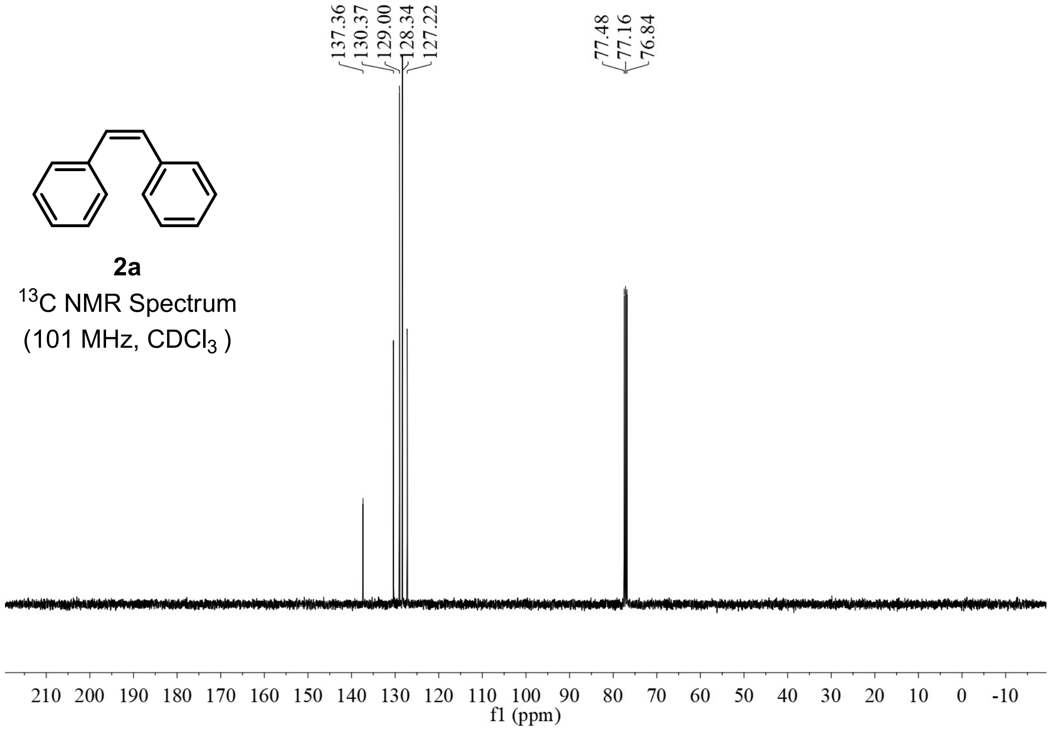

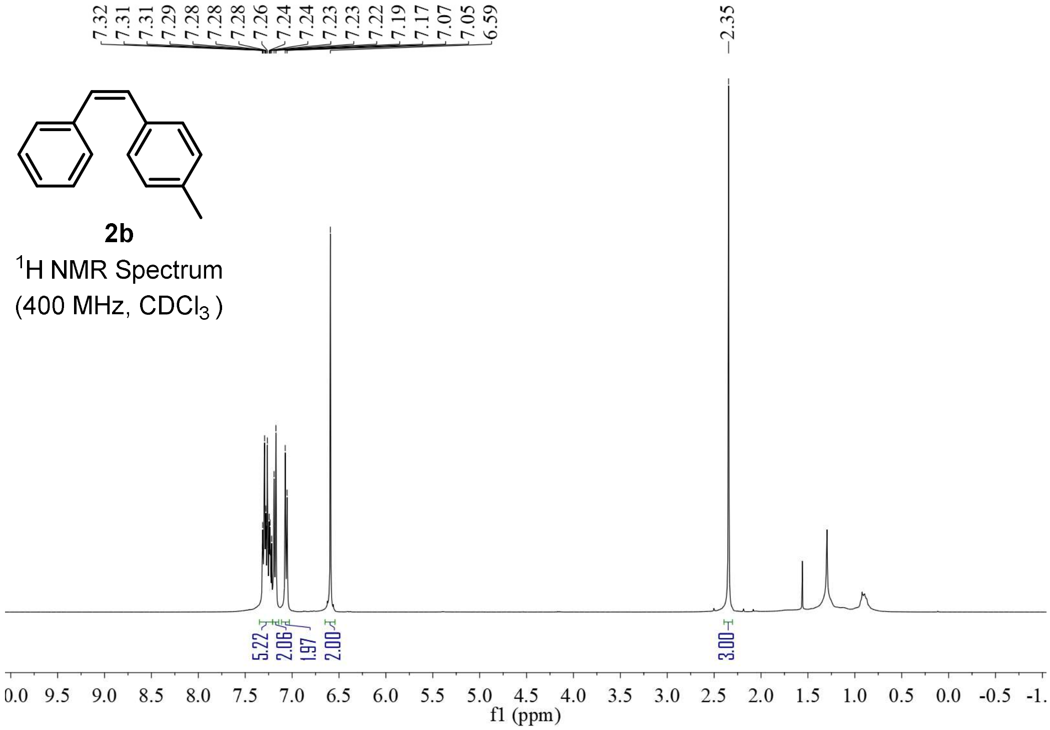
7.32
7.31
7.31
7.29
7.28
7.28
7.28
7.26
7.24
7.24
7.23
7.23
7.22
7.19
7.17
7.07
7.05
6.59
2.35
2b
1H NMR Spectrum
(400 MHz, CDCl3)
5.22
2.06
1.97
2.00
3.00
0.0 9.5 9.0 8.5 8.0 7.5 7.0 6.5 6.0 5.5 5.0 4.5 4.0 3.5 3.0 2.5 2.0 1.5 1.0 0.5 0.0 -0.5 -1.
f1 (ppm)

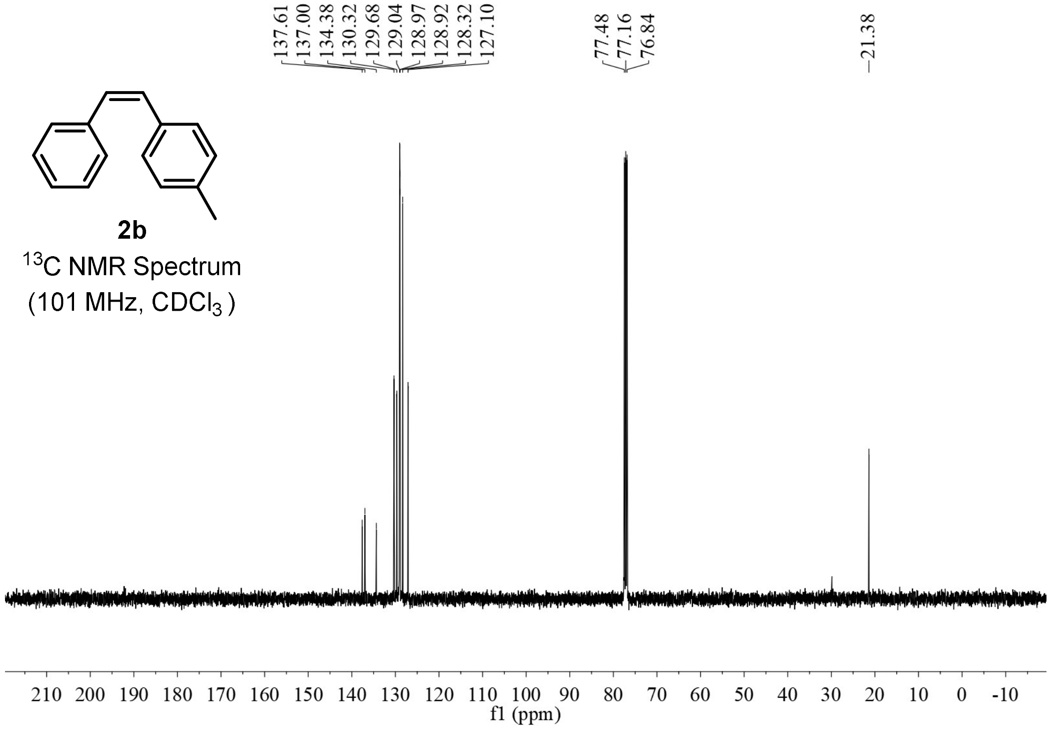
137.61
137.00
134.38
130.32
129.68
129.04
128.97
128.92
128.32
127.10
77.48
77.16
76.84
21.38
2b
13C NMR Spectrum
(101 MHz, CDCl3)
210 200 190 180 170 160 150 140 130 120 110 100 90 80 70 60 50 40 30 20 10 0 -10
f1 (ppm)

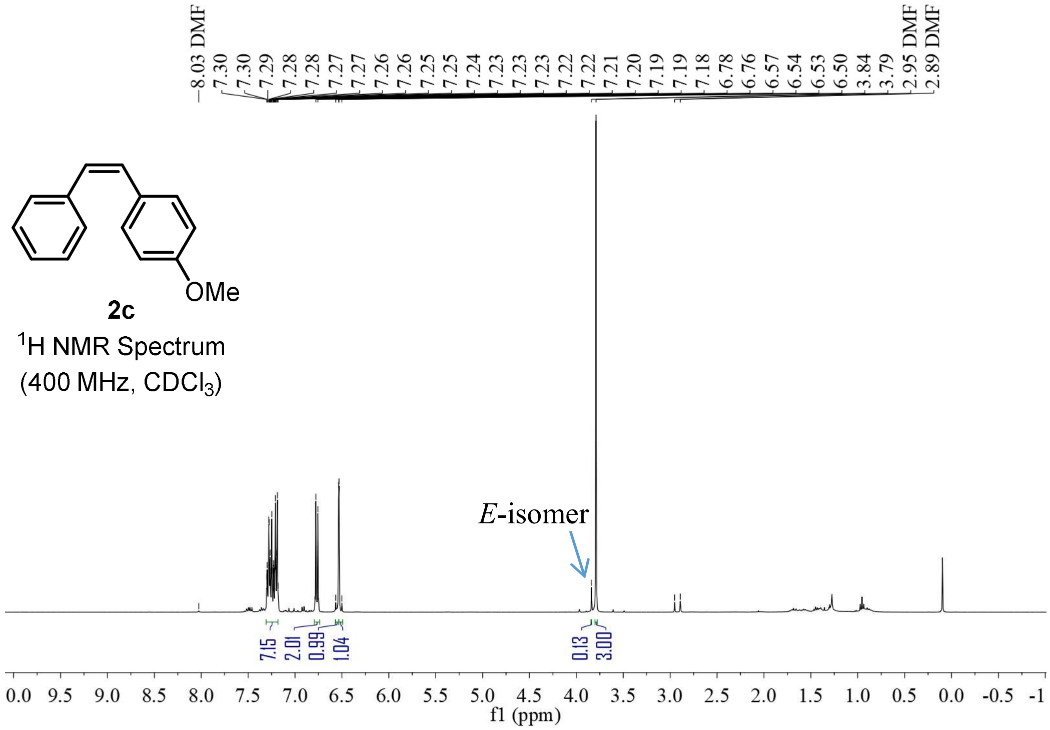

8.03 DMF
7.30
7.30
7.29
7.28
7.28
7.27
7.27
7.26
7.26
7.25
7.25
7.24
7.23
7.23
7.23
7.22
7.22
7.21
7.20
7.19
7.19
7.18
6.78
6.76
6.57
6.54
6.53
6.50
3.84
3.79
2.95 DMF
2.89 DMF
OMe
2c
^{1}H NMR Spectrum
(400 MHz, $CDCl_3$)
E-isomer
7.15
2.01
0.99
1.04
0.13
3.00
0.0 9.5 9.0 8.5 8.0 7.5 7.0 6.5 6.0 5.5 5.0 4.5 4.0 3.5 3.0 2.5 2.0 1.5 1.0 0.5 0.0 -0.5 -1
f1 (ppm)

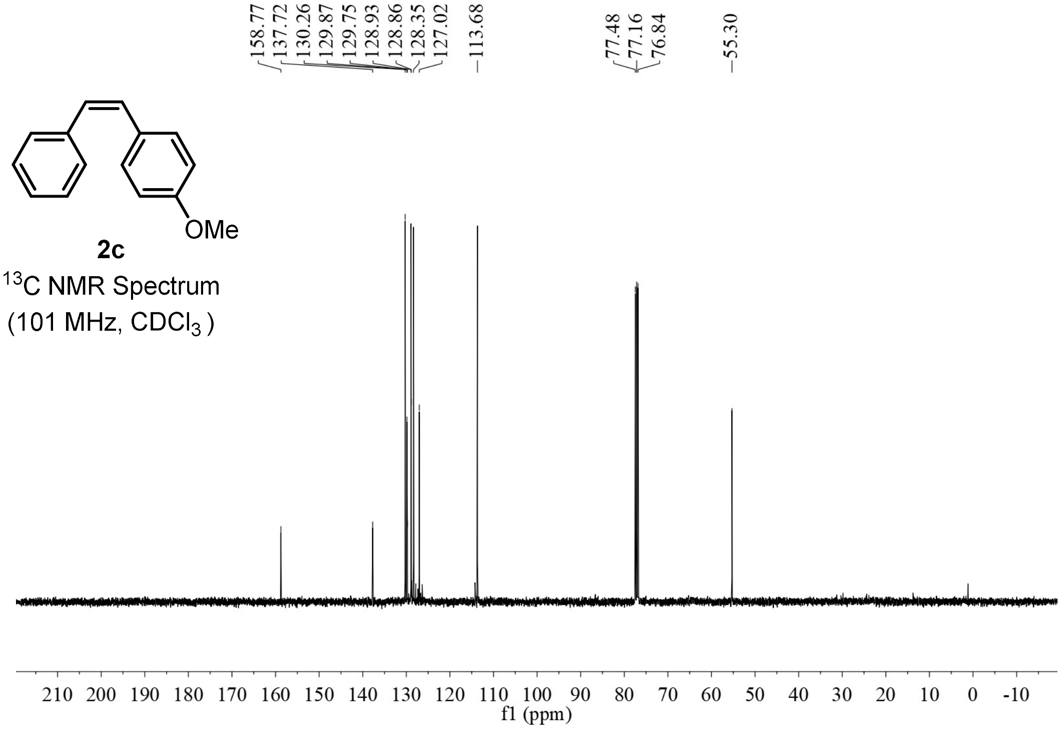

158.77
137.72
130.26
129.87
129.75
128.93
128.86
128.35
127.02
113.68
77.48
77.16
76.84
55.30
OMe
2c
^{13}C NMR Spectrum
(101 MHz, $CDCl_3$)
210 200 190 180 170 160 150 140 130 120 110 100 90 80 70 60 50 40 30 20 10 0 -10
f1 (ppm)

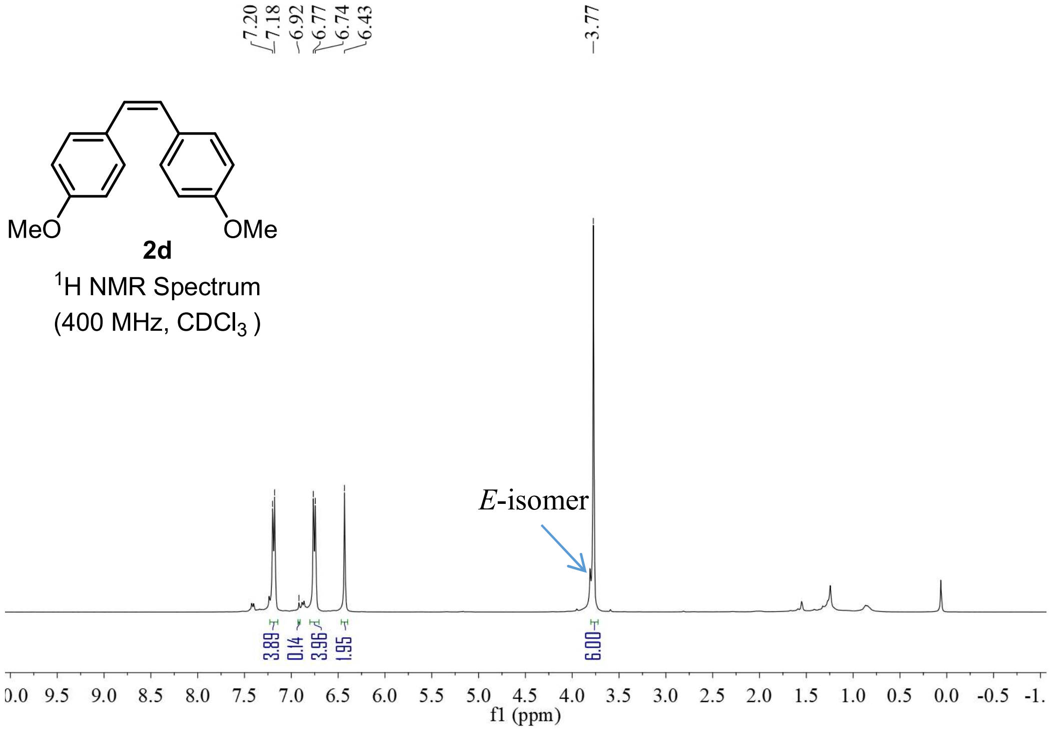
7.20
7.18
6.92
6.77
6.74
6.43
3.77
MeO
OMe
2d
¹H NMR Spectrum
(400 MHz, CDCl3)
E-isomer
3.89
0.14
3.96
1.95
6.00
f1 (ppm)

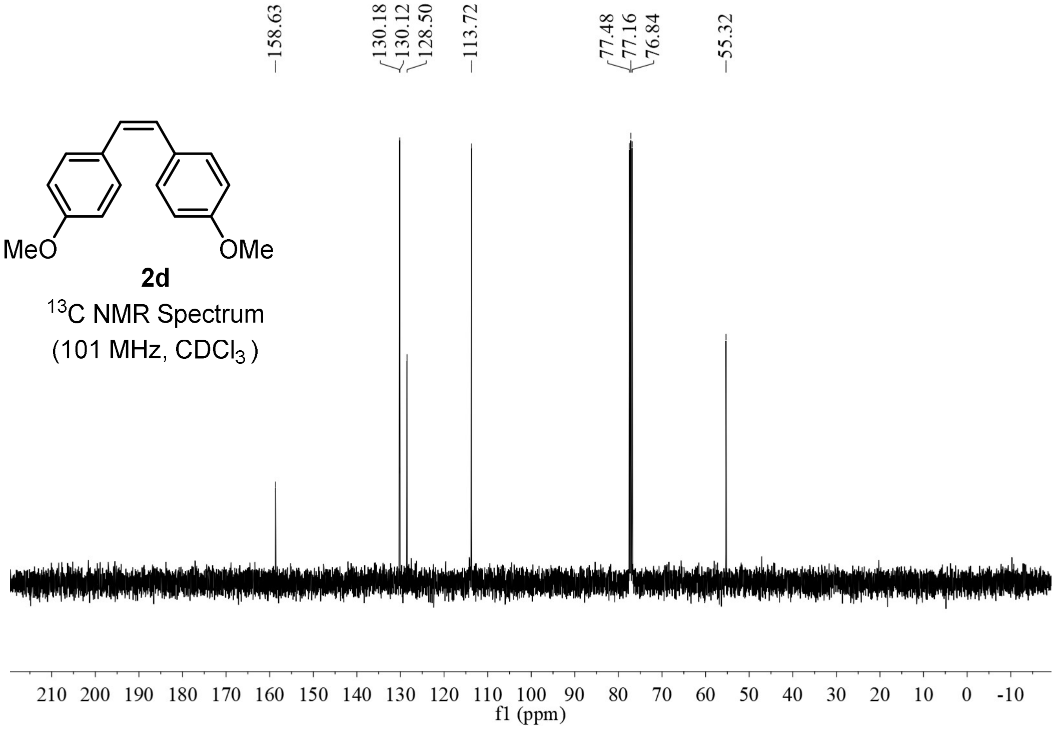
158.63
130.18
130.12
128.50
113.72
77.48
77.16
76.84
55.32
MeO
OMe
2d
¹³C NMR Spectrum
(101 MHz, CDCl3)
f1 (ppm)

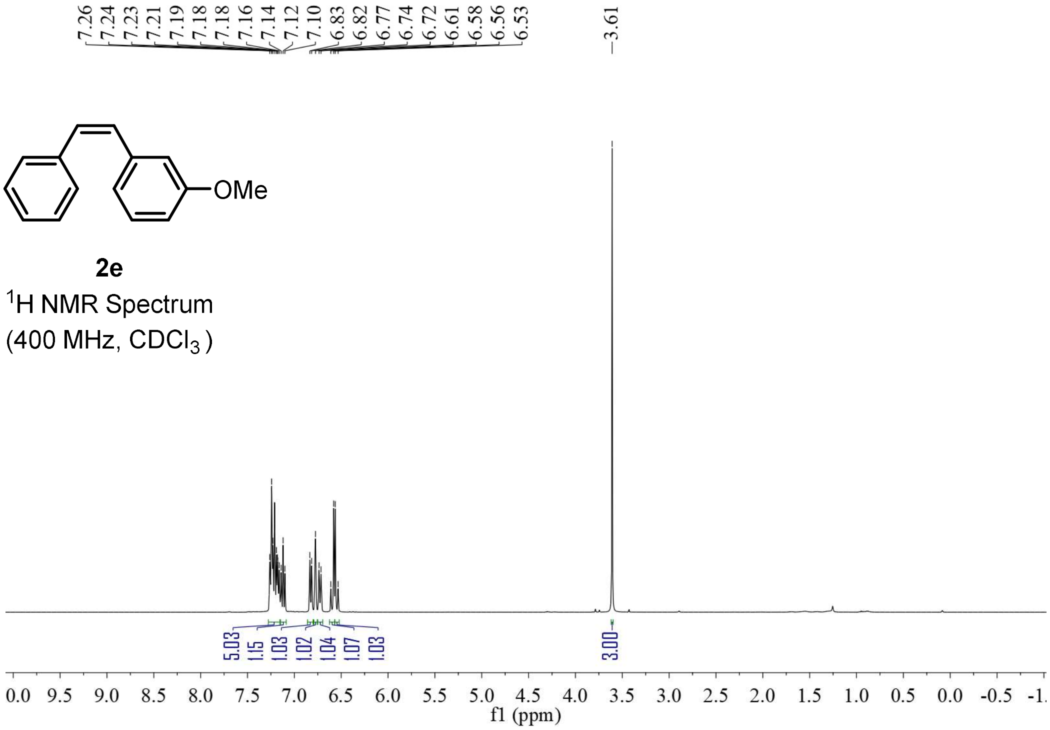
7.26 7.24 7.23 7.21 7.19 7.18 7.18 7.16 7.14 7.12 7.10 6.83 6.82 6.77 6.74 6.72 6.61 6.58 6.56 6.53
3.61
OMe
2e
1H NMR Spectrum
(400 MHz, CDCl3)
5.03 1.15 1.03 1.02 1.04 1.07 1.03
3.00
0.0 9.5 9.0 8.5 8.0 7.5 7.0 6.5 6.0 5.5 5.0 4.5 4.0 3.5 3.0 2.5 2.0 1.5 1.0 0.5 0.0 -0.5 -1.
f1 (ppm)

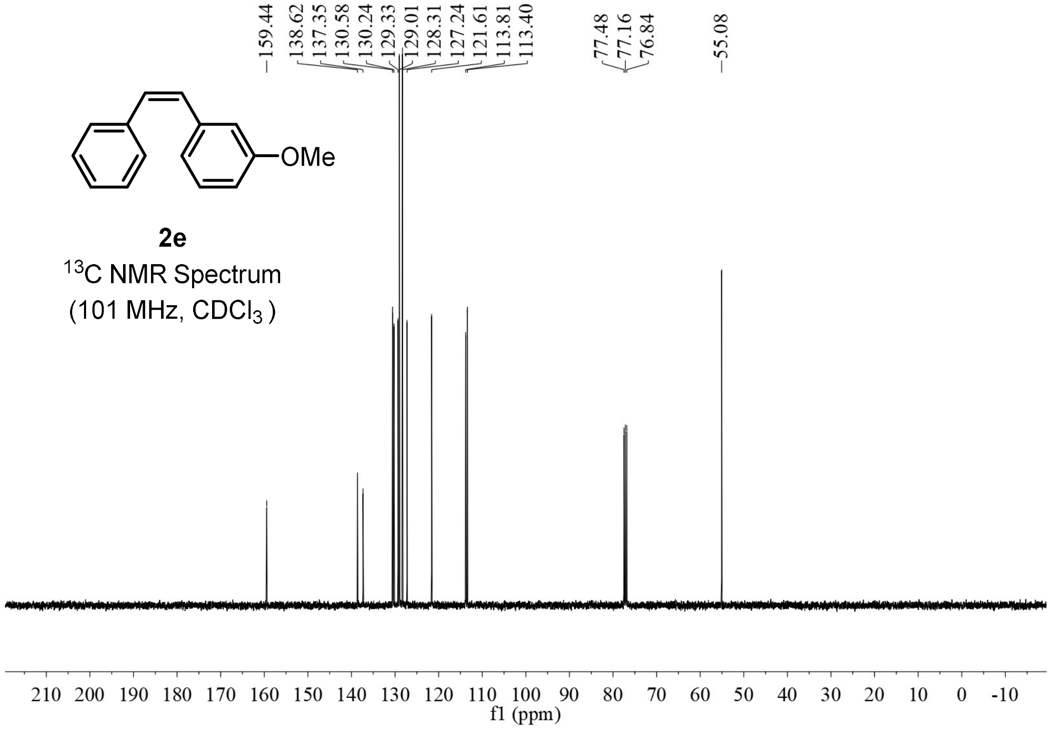
159.44
138.62 137.35 130.58 130.24 129.33 129.01 128.31 127.24 121.61 113.81 113.40
77.48 77.16 76.84
55.08
OMe
2e
13C NMR Spectrum
(101 MHz, CDCl3)
210 200 190 180 170 160 150 140 130 120 110 100 90 80 70 60 50 40 30 20 10 0 -10
f1 (ppm)

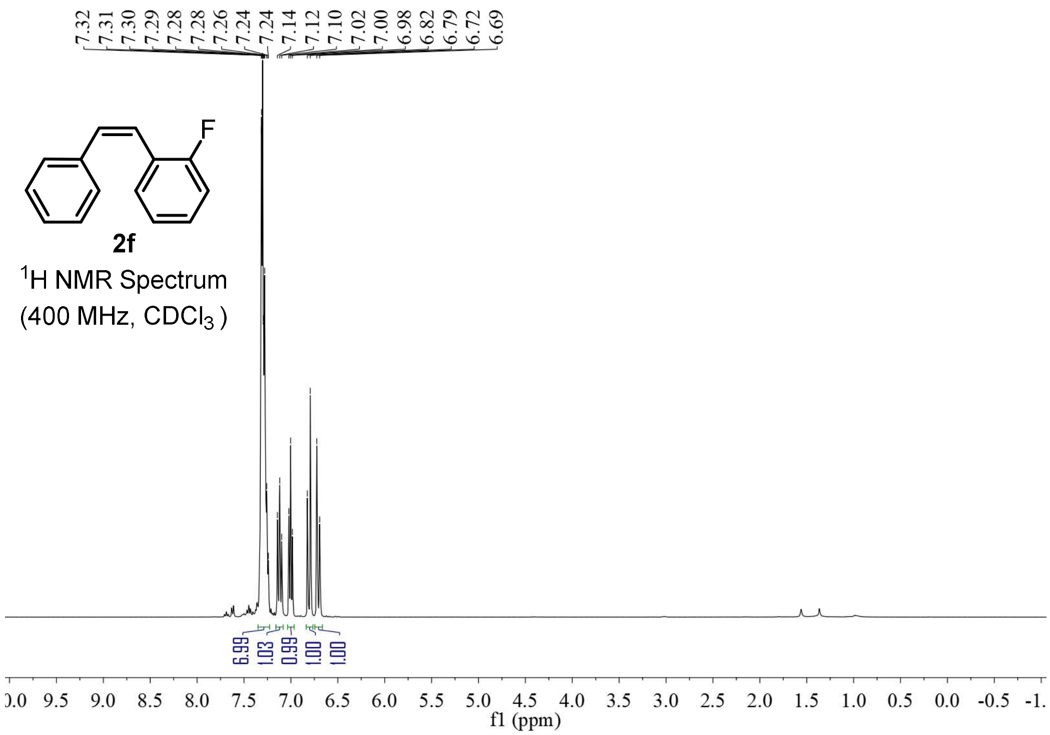
7.32
7.31
7.30
7.29
7.28
7.28
7.26
7.24
7.24
7.14
7.12
7.10
7.02
7.00
6.98
6.82
6.79
6.72
6.69
F
2f
^{1}H NMR Spectrum
(400 MHz, $CDCl_3$)
6.99
1.03
0.99
1.00
1.00
f1 (ppm)

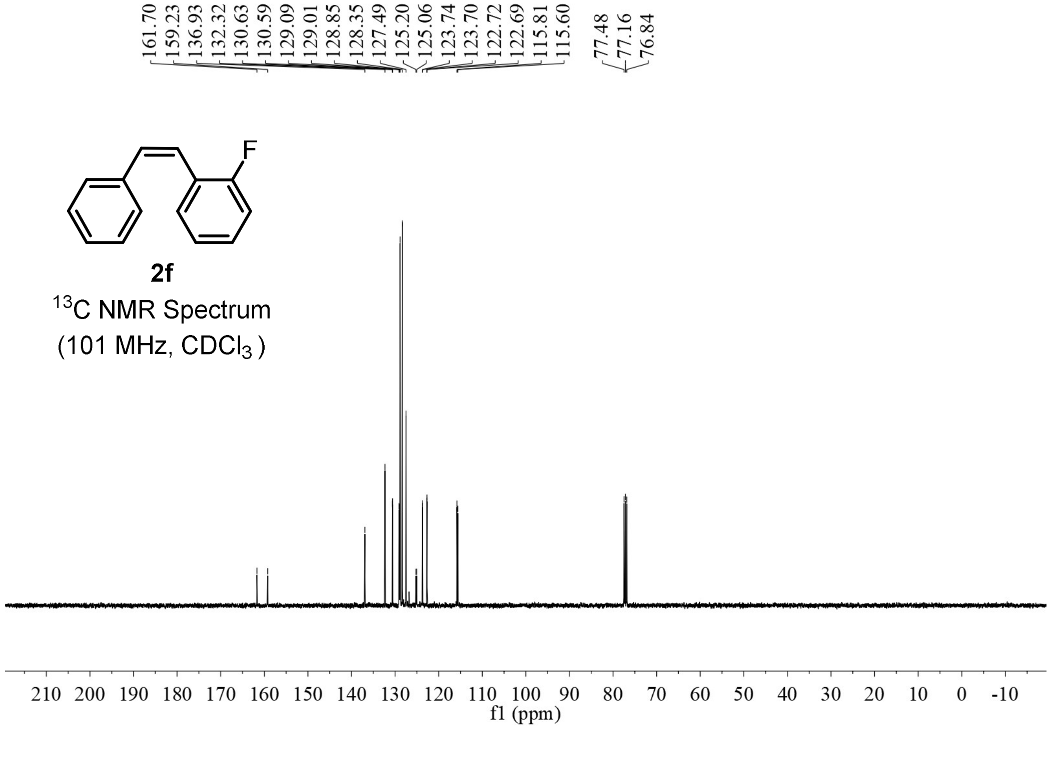
161.70
159.23
136.93
132.32
130.63
130.59
129.09
129.01
128.85
128.35
127.49
125.20
125.06
123.74
123.70
122.72
122.69
115.81
115.60
77.48
77.16
76.84
F
2f
^{13}C NMR Spectrum
(101 MHz, $CDCl_3$)
f1 (ppm)

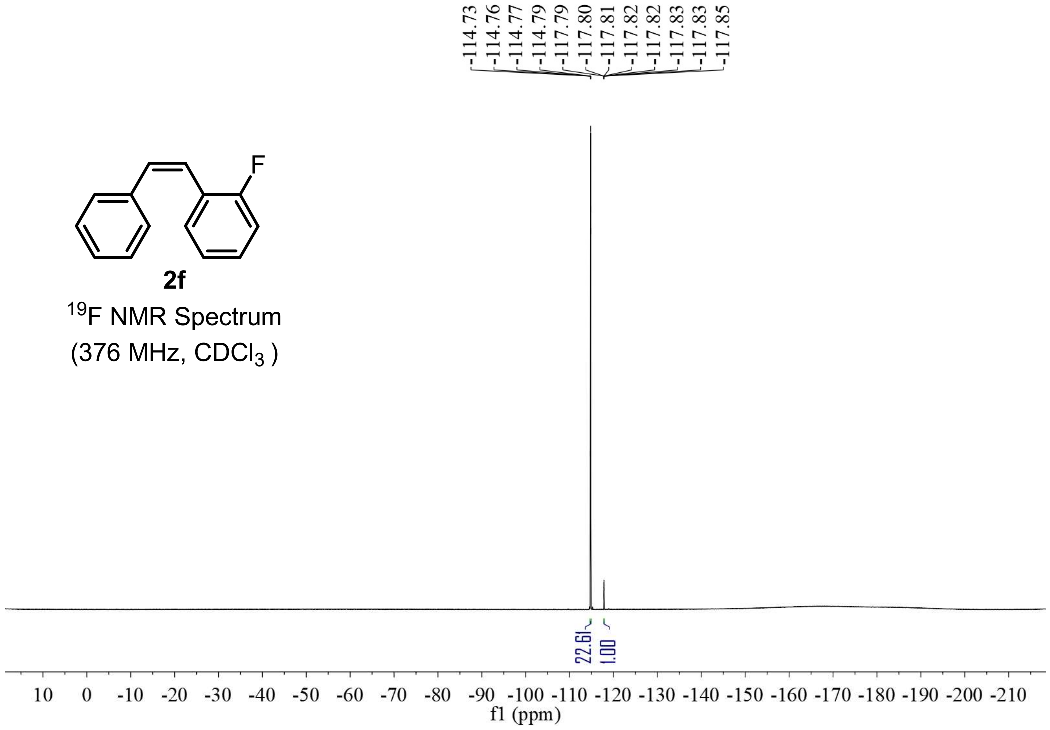

2f

^{19}F NMR Spectrum
(376 MHz, $CDCl_3$)

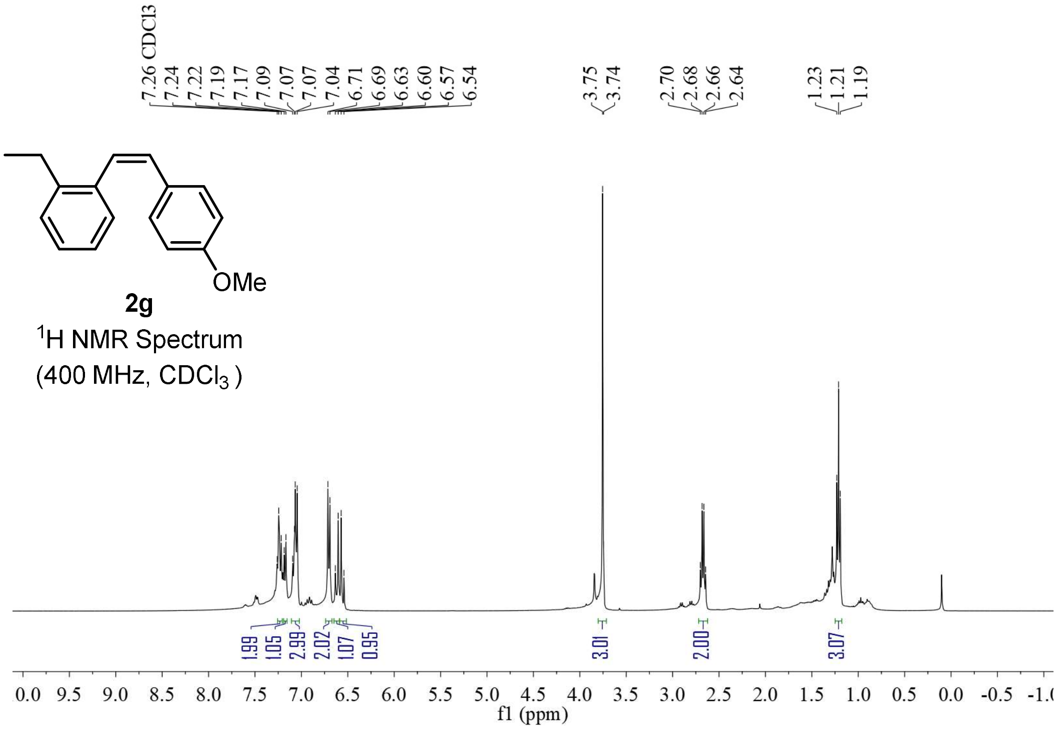

2g

^{1}H NMR Spectrum
(400 MHz, $CDCl_3$)

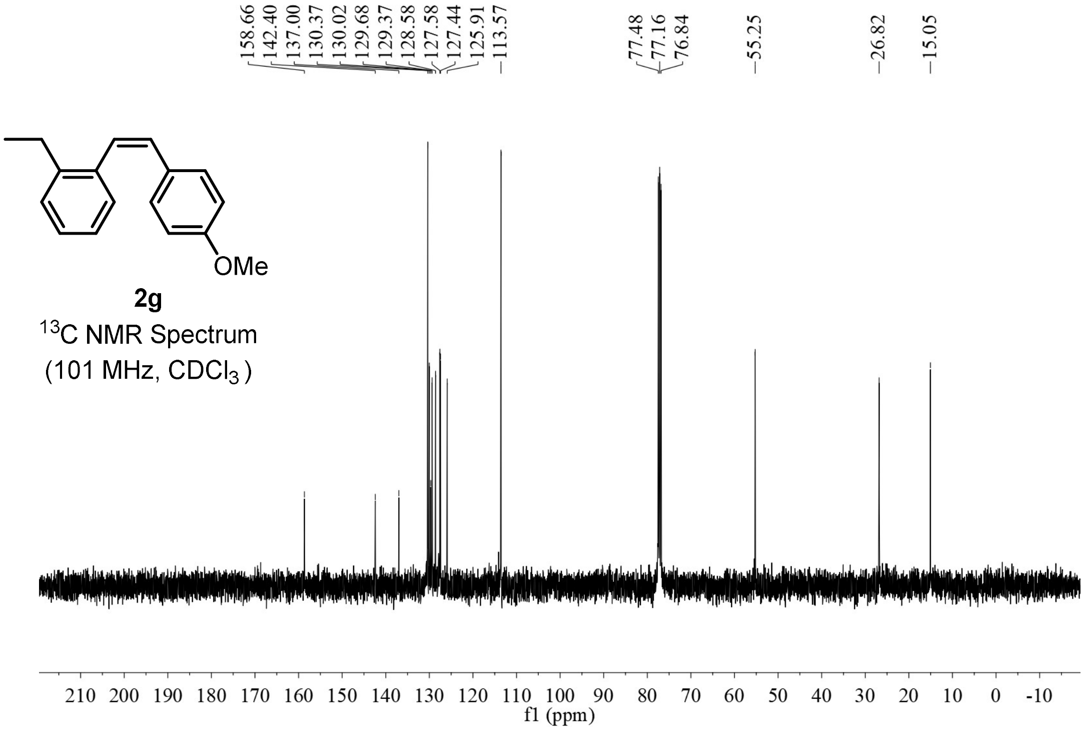
158.66
142.40
137.00
130.37
130.02
129.68
129.37
128.58
127.58
127.44
125.91
113.57
77.48
77.16
76.84
55.25
26.82
15.05
OMe
2g
^{13}C NMR Spectrum
(101 MHz, $CDCl_3$)
210 200 190 180 170 160 150 140 130 120 110 100 90 80 70 60 50 40 30 20 10 0 -10
f1 (ppm)

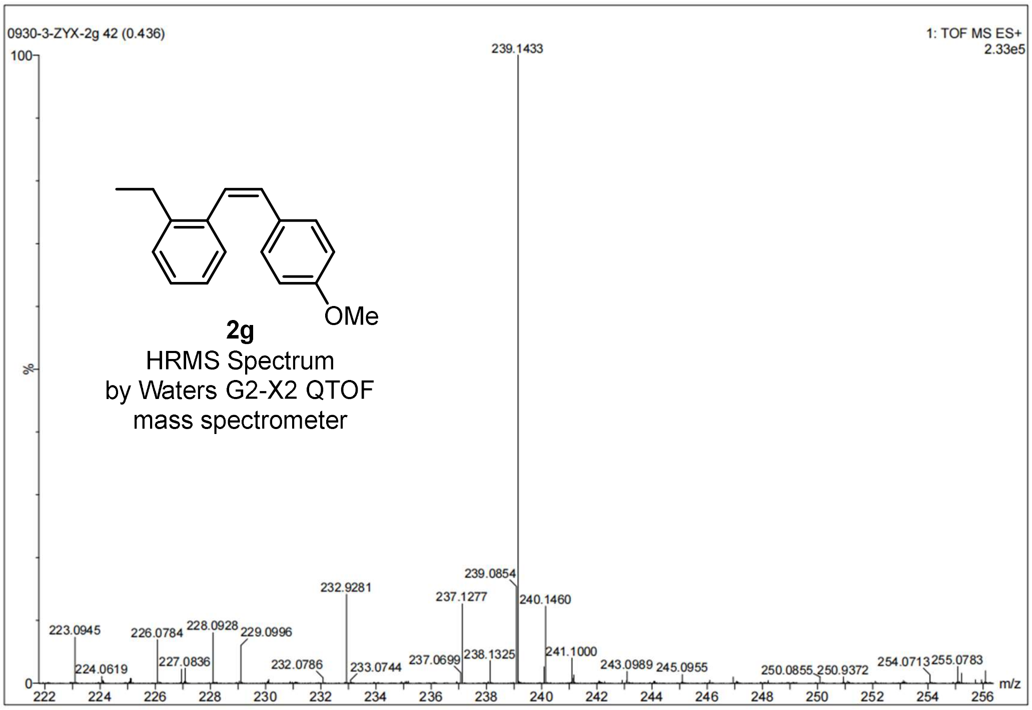
0930-3-ZYX-2g 42 (0.436)
1: TOF MS ES+
2.33e5
239.1433
OMe
2g
HRMS Spectrum
by Waters G2-X2 QTOF
mass spectrometer
%
239.0854
232.9281
237.1277
240.1460
223.0945
226.0784
228.0928
229.0996
241.1000
224.0619
227.0836
232.0786
233.0744
237.0699
238.1325
243.0989
245.0955
250.0855
250.9372
254.0713
255.0783
m/z
222 224 226 228 230 232 234 236 238 240 242 244 246 248 250 252 254 256

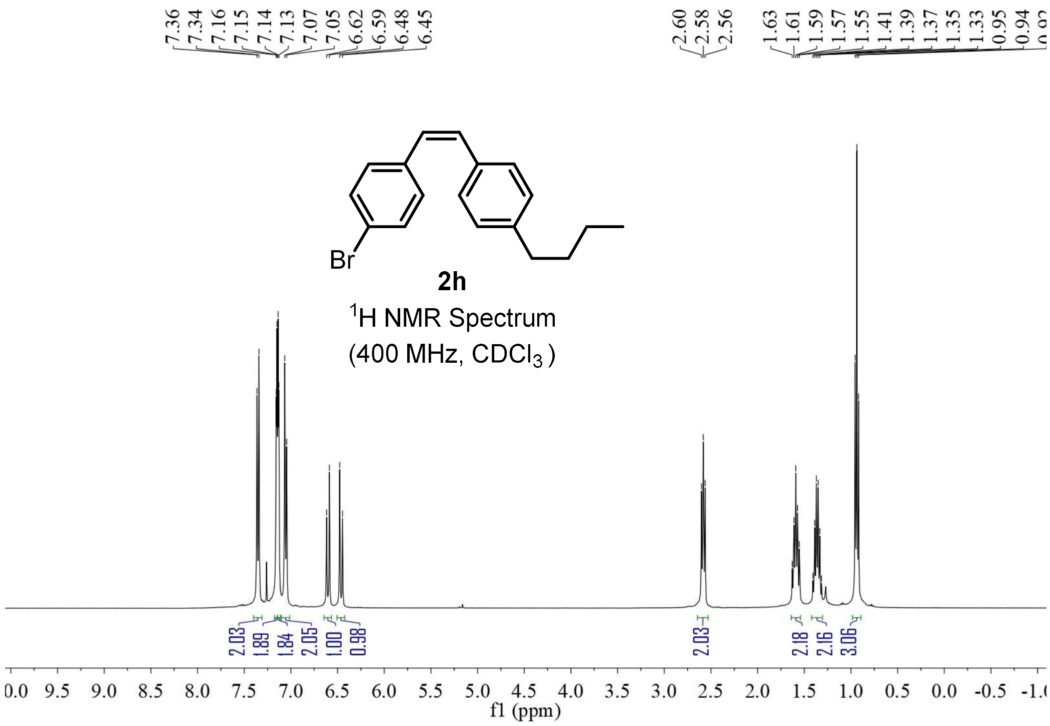
Br
2h
1H NMR Spectrum
(400 MHz, CDCl3)
f1 (ppm)

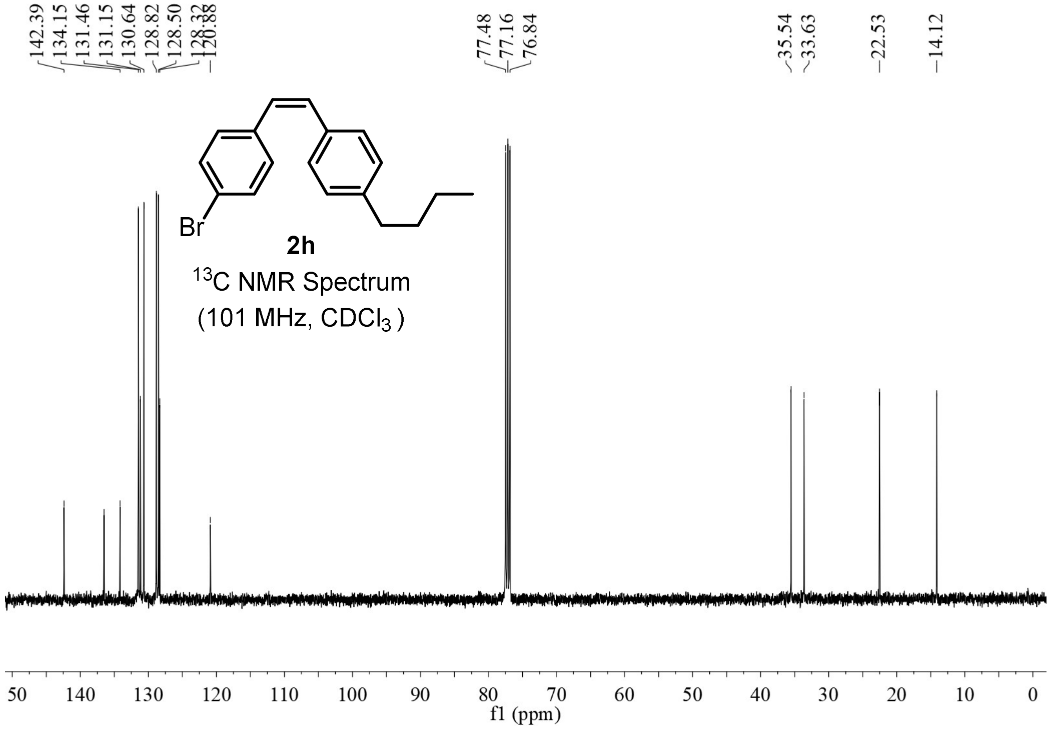
Br
2h
13C NMR Spectrum
(101 MHz, CDCl3)
f1 (ppm)

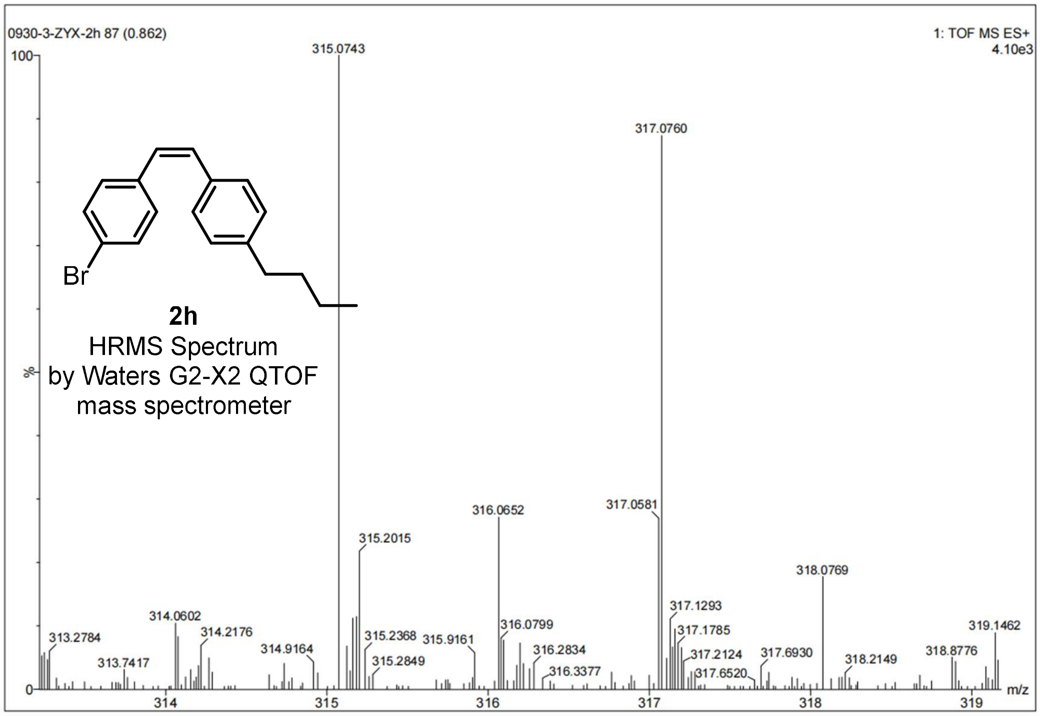
0930-3-ZYX-2h 87 (0.862)
1: TOF MS ES+
4.10e3
Br
2h
HRMS Spectrum
by Waters G2-X2 QTOF
mass spectrometer
315.0743
317.0760
317.0581
316.0652
315.2015
318.0769
314.0602
314.2176
313.2784
313.7417
314.9164
315.2368
315.2849
315.9161
316.0799
316.2834
316.3377
317.1293
317.1785
317.2124
317.6520
317.6930
318.2149
318.8776
319.1462
m/z
%

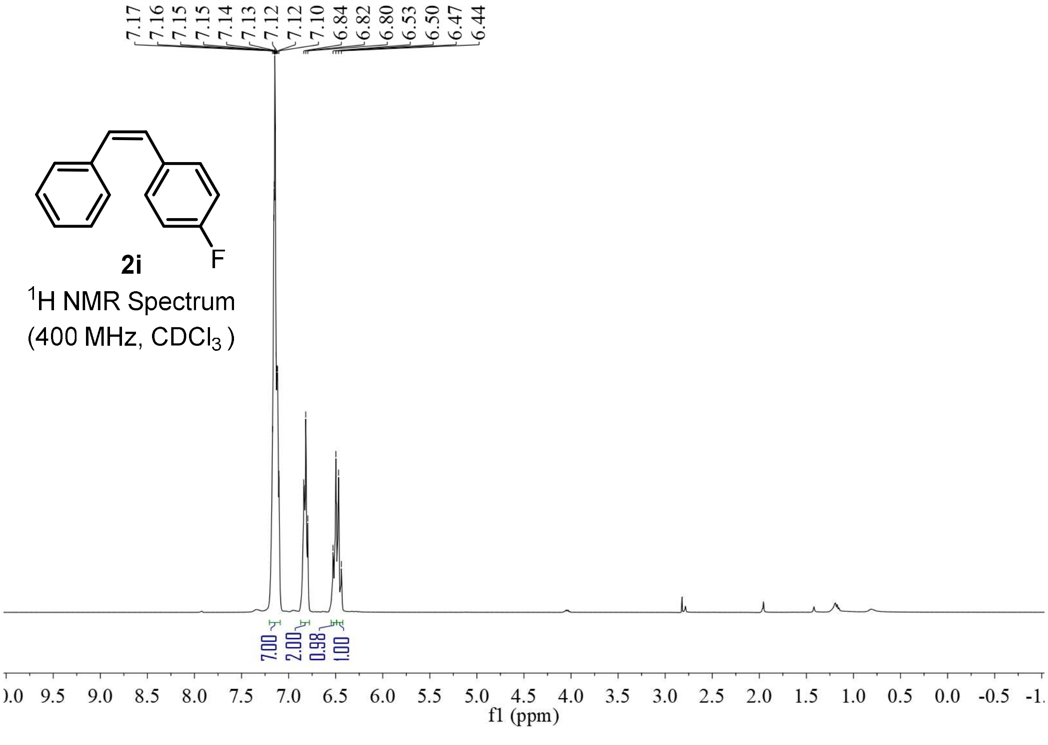
7.17
7.16
7.15
7.15
7.14
7.13
7.12
7.12
7.10
6.84
6.82
6.80
6.53
6.50
6.47
6.44
F
2i
1H NMR Spectrum
(400 MHz, CDCl3)
7.00
2.00
0.98
1.00
f1 (ppm)

2i

^{13}C NMR Spectrum
(101 MHz, $CDCl_3$)

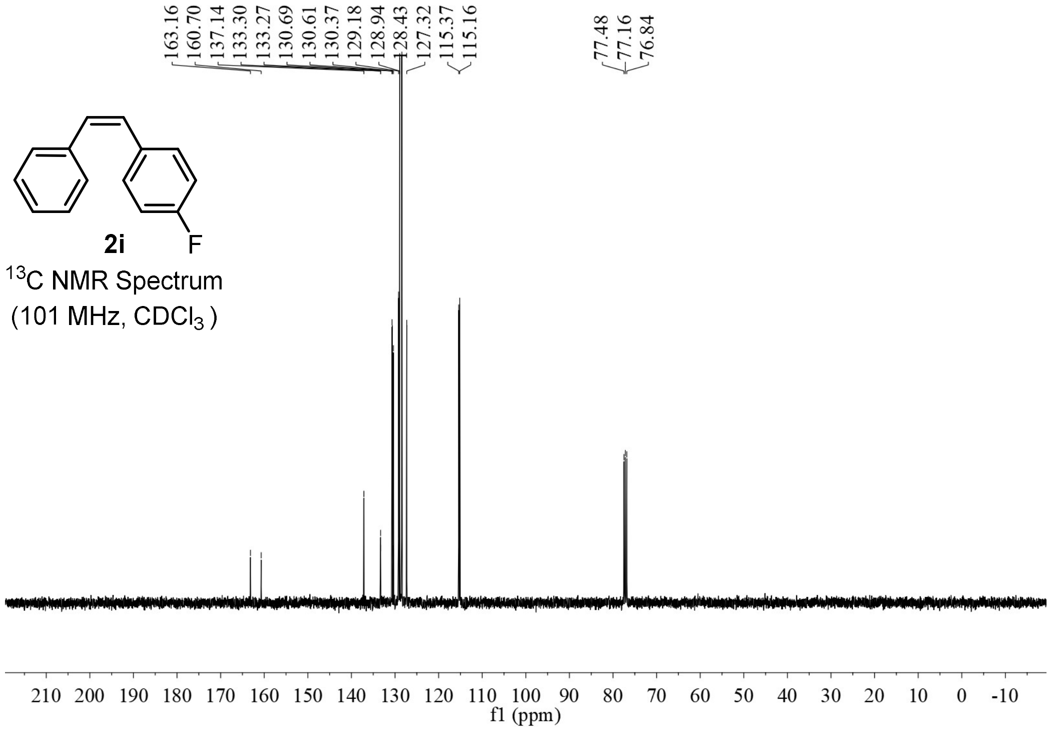

2i

^{19}F NMR Spectrum
(376 MHz, $CDCl_3$)

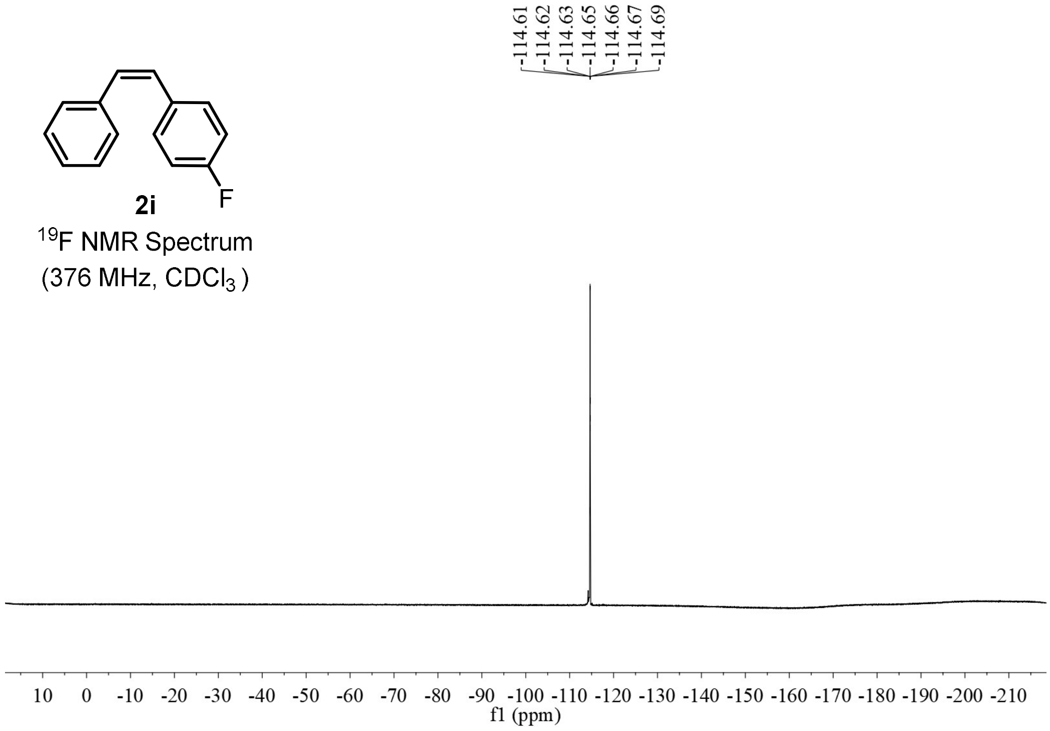

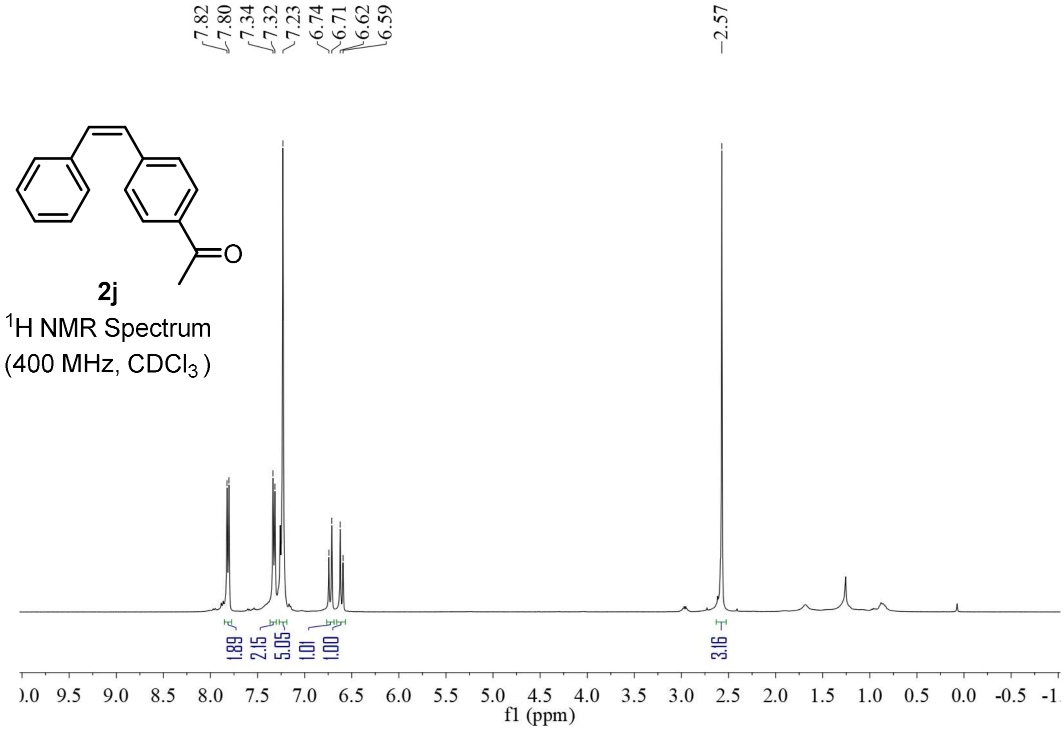
7.82
7.80
7.34
7.32
7.23
6.74
6.71
6.62
6.59
2.57
O
2j
^{1}H NMR Spectrum
(400 MHz, $CDCl_3$)
1.89
2.15
5.05
1.01
1.00
3.16
f1 (ppm)

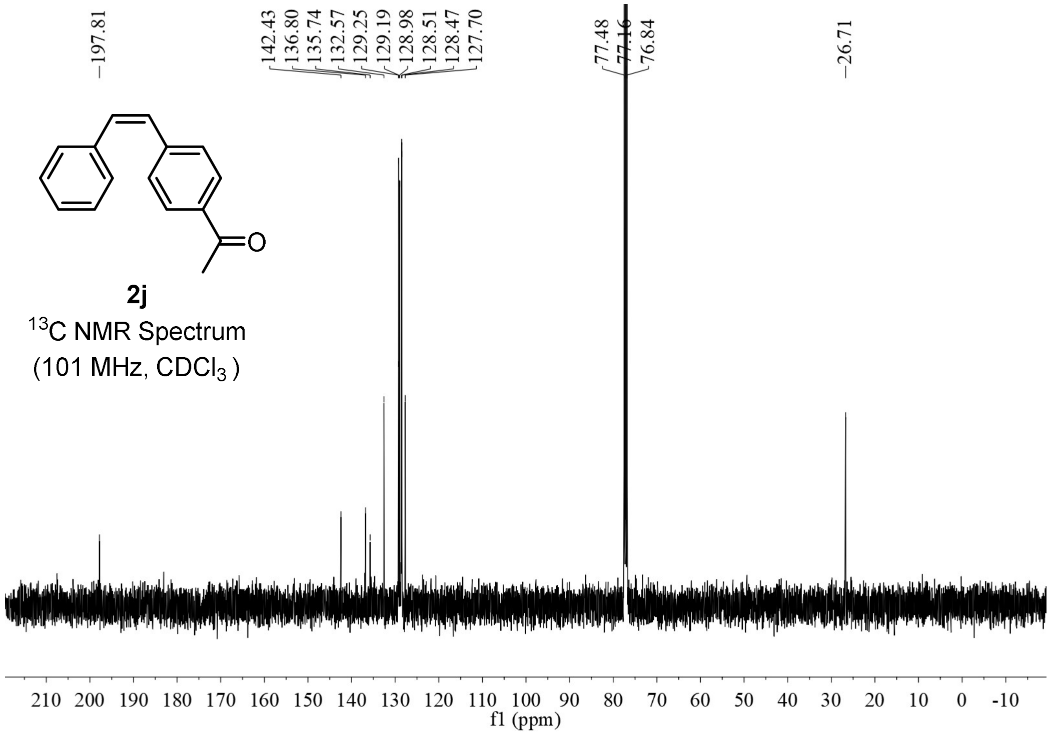
197.81
142.43
136.80
135.74
132.57
129.25
129.19
128.98
128.51
128.47
127.70
77.48
77.16
76.84
26.71
O
2j
^{13}C NMR Spectrum
(101 MHz, $CDCl_3$)
f1 (ppm)

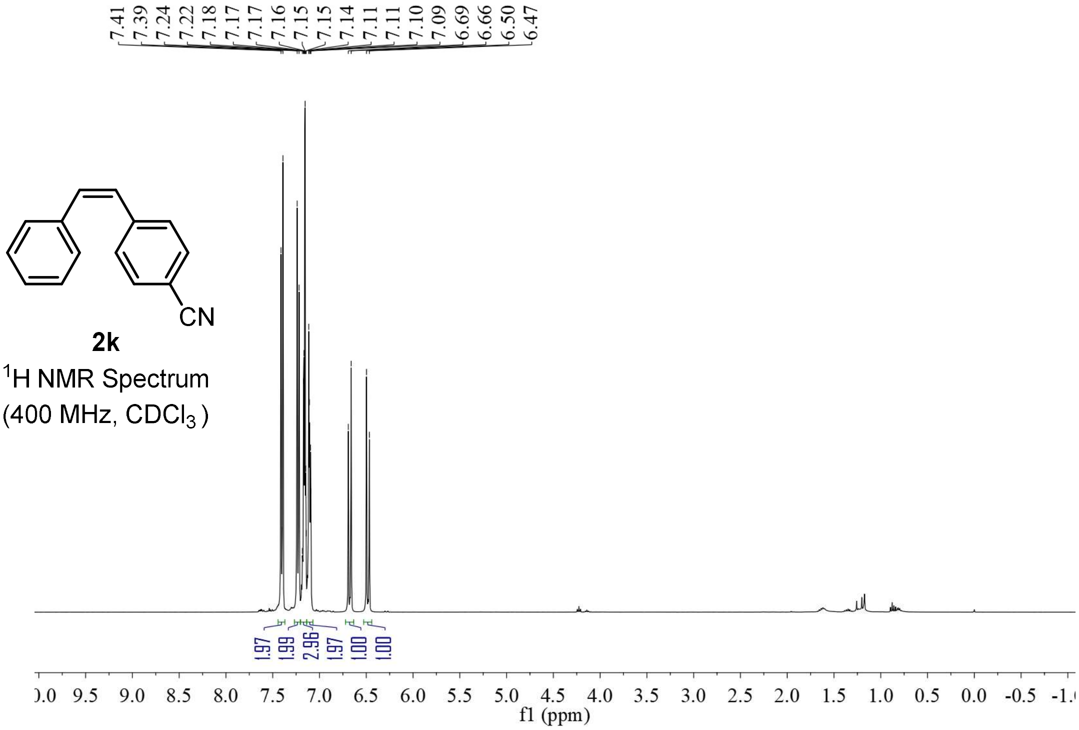
7.41 7.39 7.24 7.22 7.18 7.17 7.17 7.16 7.15 7.15 7.14 7.11 7.11 7.10 7.09 6.69 6.66 6.50 6.47
CN
2k
^{1}H NMR Spectrum
(400 MHz, $CDCl_3$)
1.97 1.99 2.96 1.97 1.00 1.00
f1 (ppm)

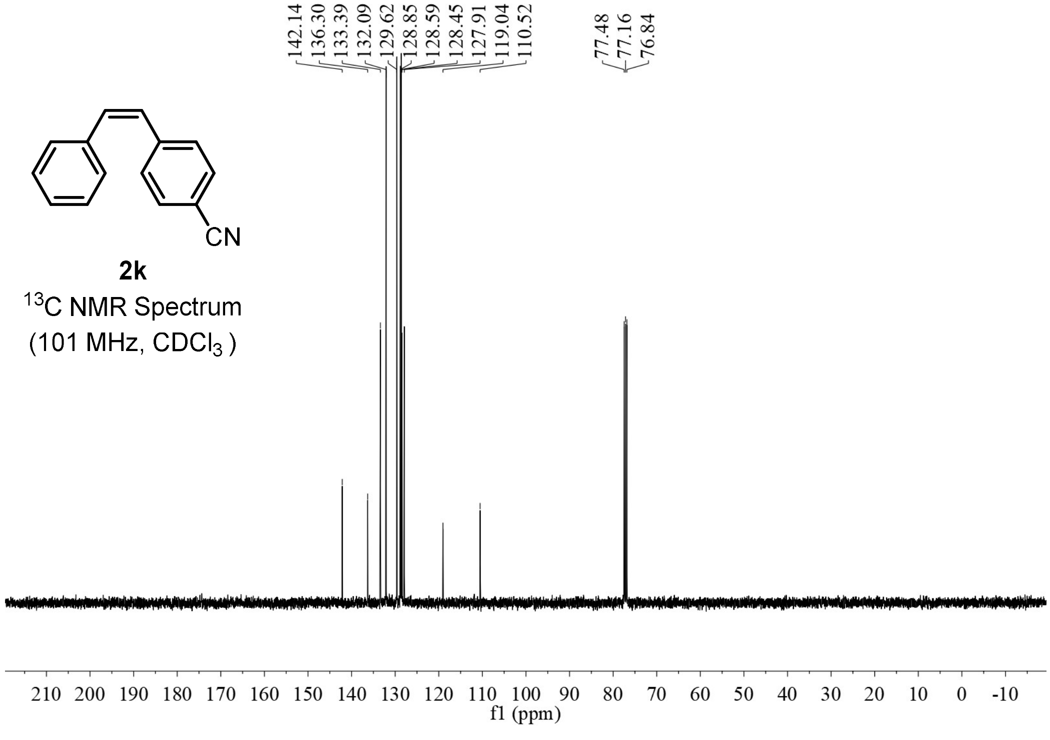
142.14 136.30 133.39 132.09 129.62 128.85 128.59 128.45 127.91 119.04 110.52
77.48 77.16 76.84
CN
2k
^{13}C NMR Spectrum
(101 MHz, $CDCl_3$)
210 200 190 180 170 160 150 140 130 120 110 100 90 80 70 60 50 40 30 20 10 0 -10
f1 (ppm)

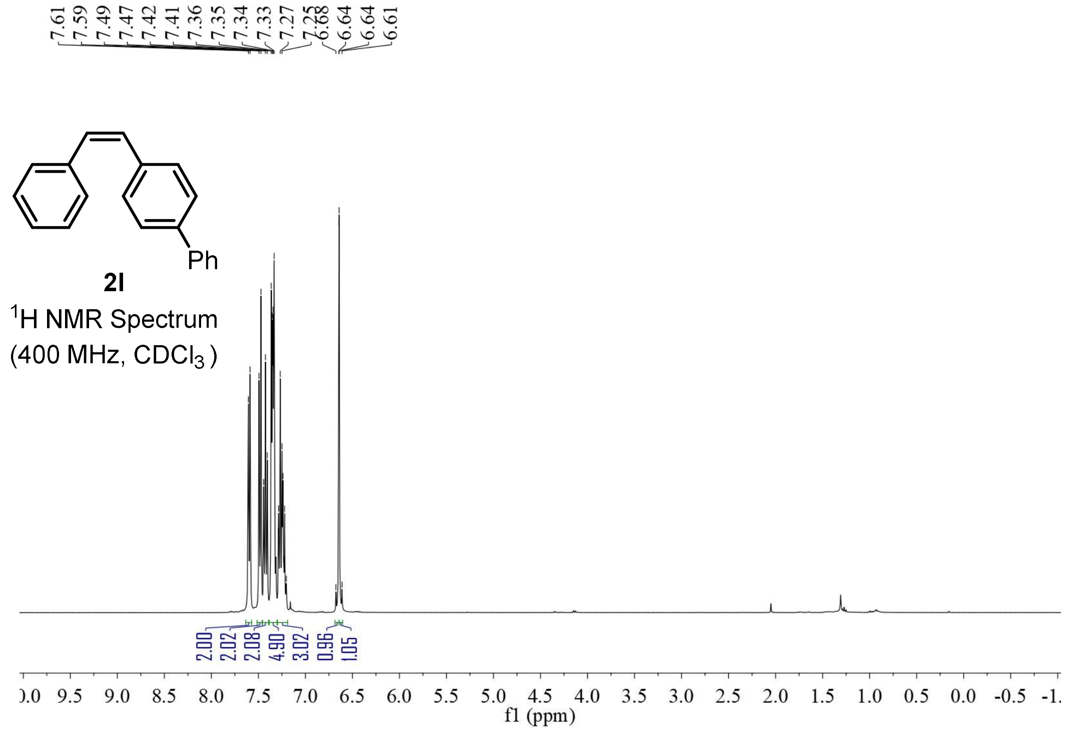
7.61
7.59
7.49
7.47
7.42
7.41
7.36
7.35
7.34
7.33
7.27
7.25
6.68
6.64
6.64
6.61
Ph
2l
1H NMR Spectrum
(400 MHz, CDCl3)
2.00
2.02
2.08
4.90
3.02
0.96
1.05
f1 (ppm)

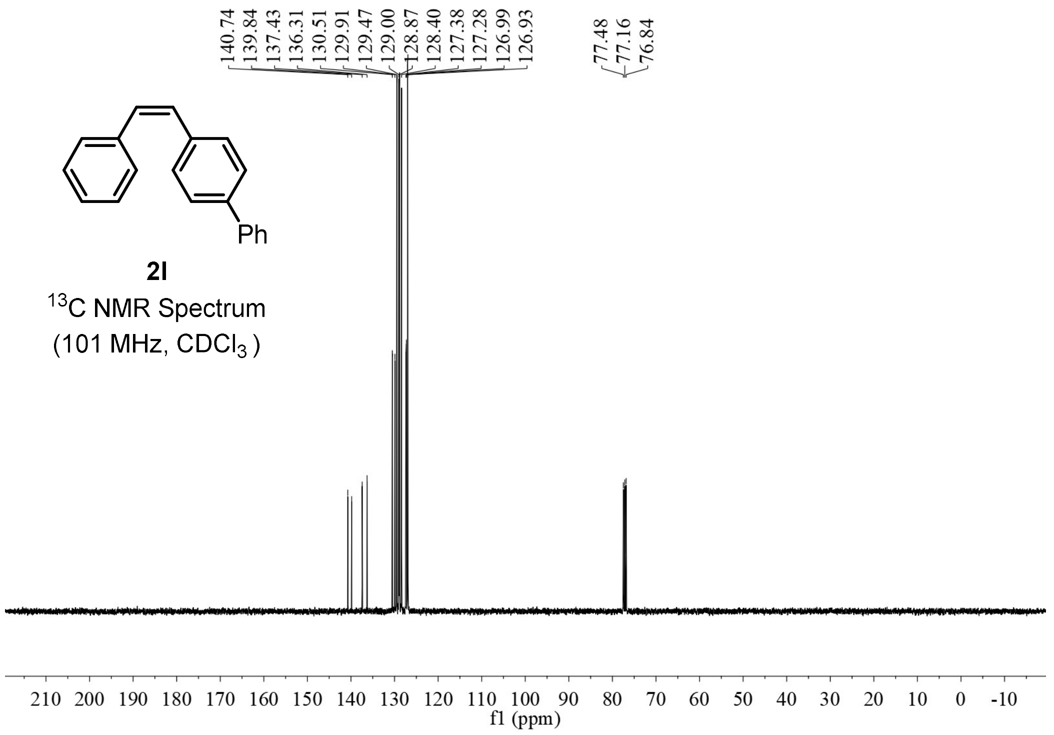
140.74
139.84
137.43
136.31
130.51
129.91
129.47
129.00
128.87
128.40
127.38
127.28
126.99
126.93
77.48
77.16
76.84
Ph
2l
13C NMR Spectrum
(101 MHz, CDCl3)
f1 (ppm)

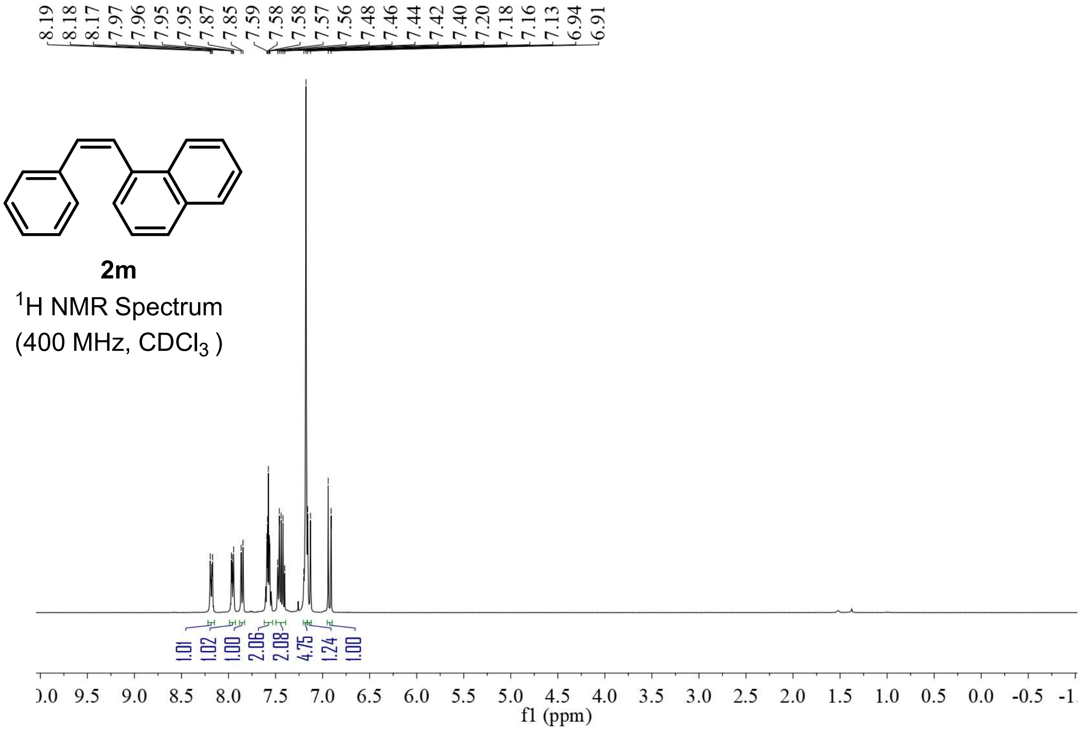
8.19 8.18 8.17 7.97 7.96 7.95 7.95 7.87 7.85 7.59 7.58 7.58 7.57 7.56 7.48 7.46 7.44 7.42 7.40 7.20 7.18 7.16 7.13 6.94 6.91
2m
^{1}H NMR Spectrum
(400 MHz, $CDCl_3$)
1.01 1.02 1.00 2.06 2.08 4.75 1.24 1.00
f1 (ppm)

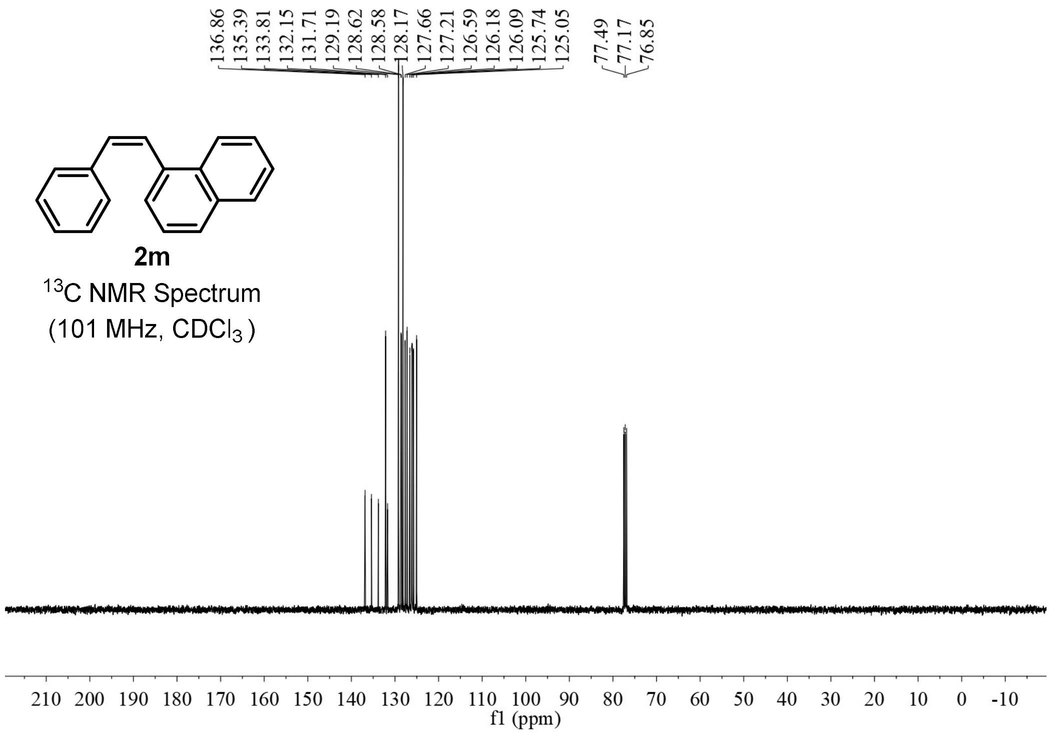
136.86 135.39 133.81 132.15 131.71 129.19 128.62 128.58 128.17 127.66 127.21 126.59 126.18 126.09 125.74 125.05
77.49 77.17 76.85
2m
^{13}C NMR Spectrum
(101 MHz, $CDCl_3$)
f1 (ppm)

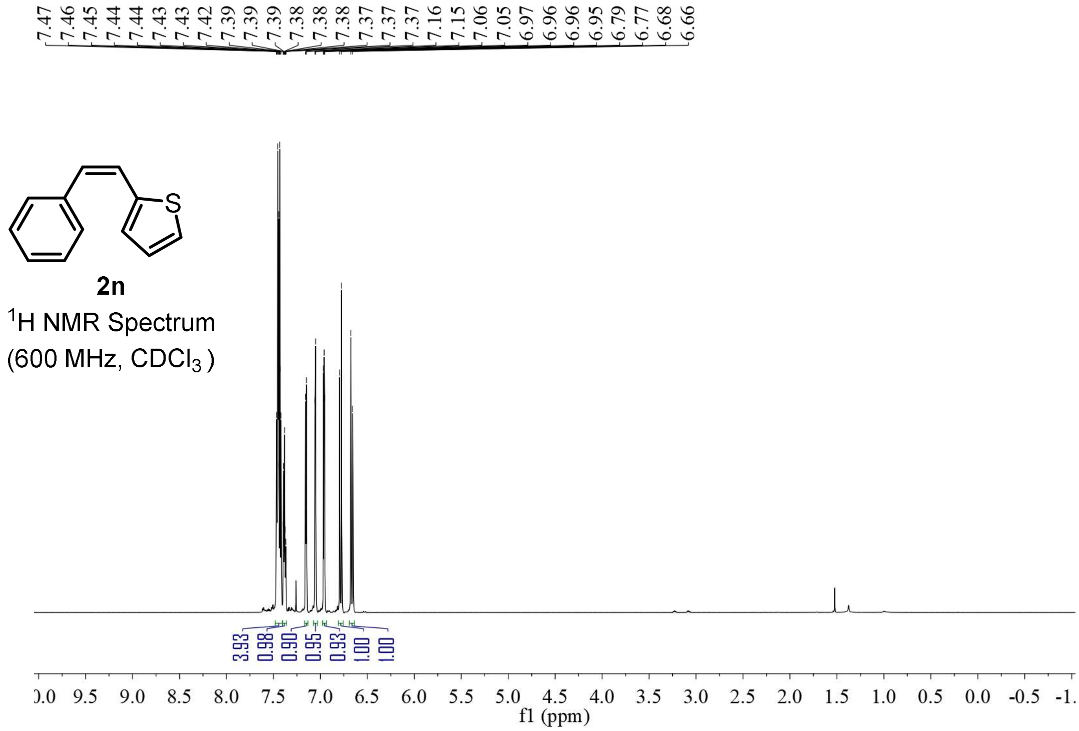
7.47
7.46
7.45
7.44
7.44
7.43
7.43
7.42
7.39
7.39
7.39
7.38
7.38
7.38
7.37
7.37
7.37
7.16
7.15
7.06
7.05
6.97
6.96
6.96
6.95
6.79
6.77
6.68
6.66
S
2n
1H NMR Spectrum
(600 MHz, CDCl3)
3.93
0.98
0.90
0.95
0.93
1.00
1.00
f1 (ppm)

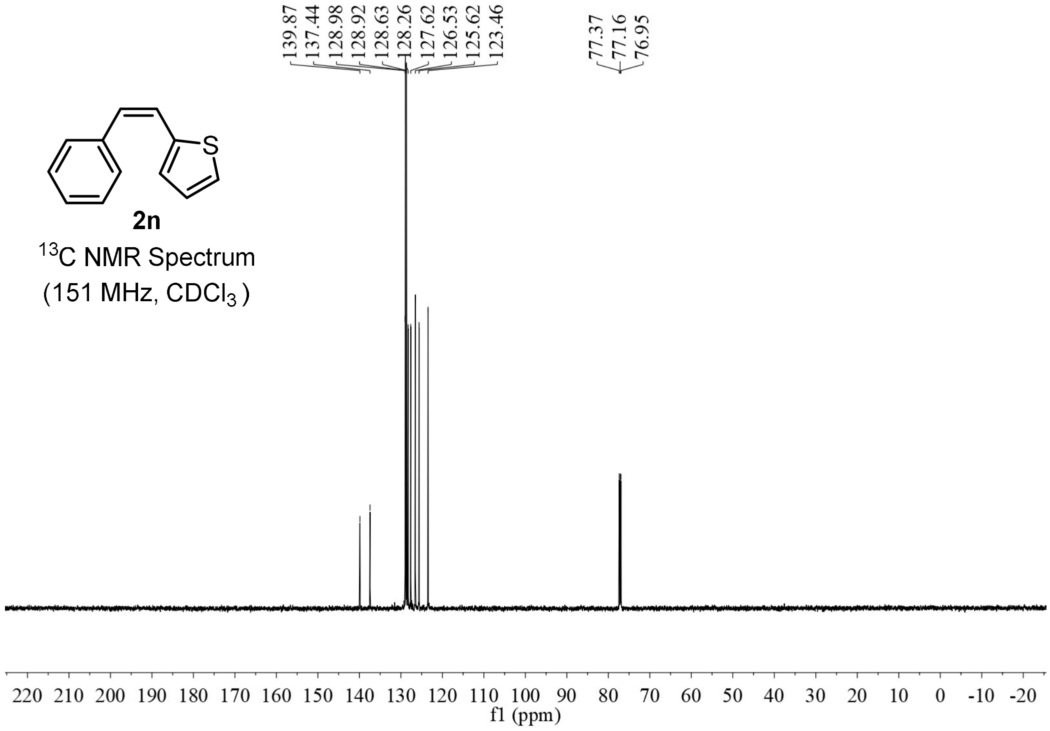
139.87
137.44
128.98
128.92
128.63
128.26
127.62
126.53
125.62
123.46
77.37
77.16
76.95
S
2n
13C NMR Spectrum
(151 MHz, CDCl3)
f1 (ppm)

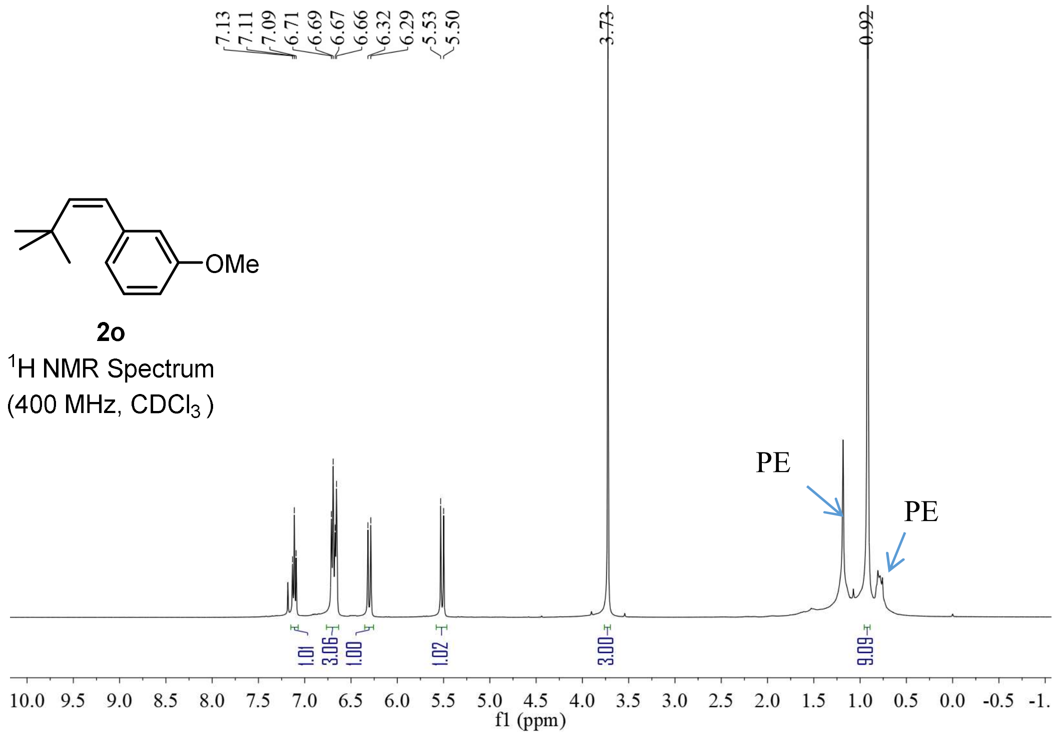
7.13
7.11
7.09
6.71
6.69
6.67
6.66
6.32
6.29
5.53
5.50
3.73
0.92
OMe
2o
^{1}H NMR Spectrum
(400 MHz, $CDCl_3$)
PE
PE
1.01
3.06
1.00
1.02
3.00
9.09
f1 (ppm)

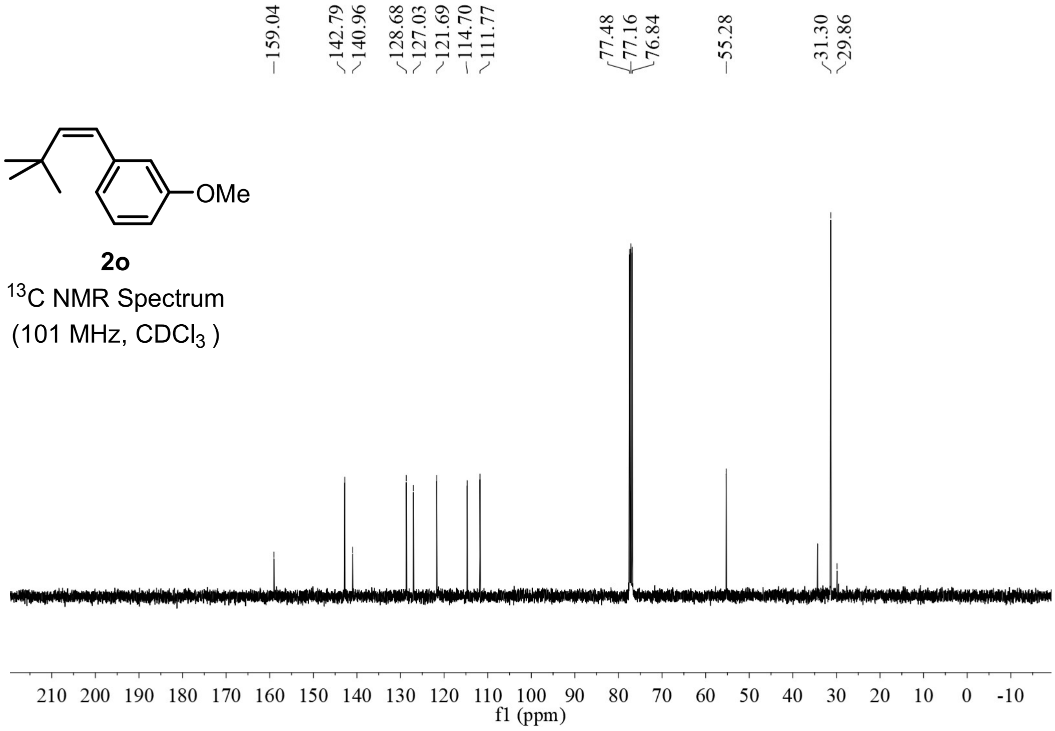
159.04
142.79
140.96
128.68
127.03
121.69
114.70
111.77
77.48
77.16
76.84
55.28
31.30
29.86
OMe
2o
^{13}C NMR Spectrum
(101 MHz, $CDCl_3$)
f1 (ppm)

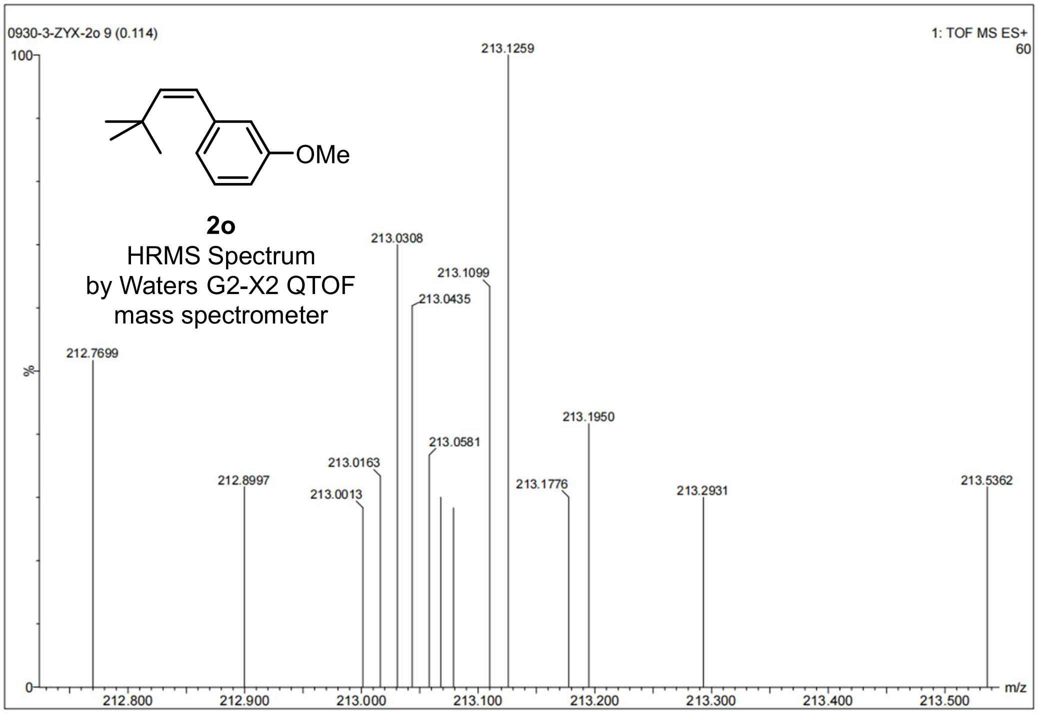
0930-3-ZYX-2o 9 (0.114)
1: TOF MS ES+
60
OMe
2o
HRMS Spectrum
by Waters G2-X2 QTOF
mass spectrometer
213.1259
213.0308
213.1099
213.0435
212.7699
213.1950
213.0581
213.0163
212.8997
213.0013
213.1776
213.2931
213.5362
100
%
0
212.800
212.900
213.000
213.100
213.200
213.300
213.400
213.500
m/z

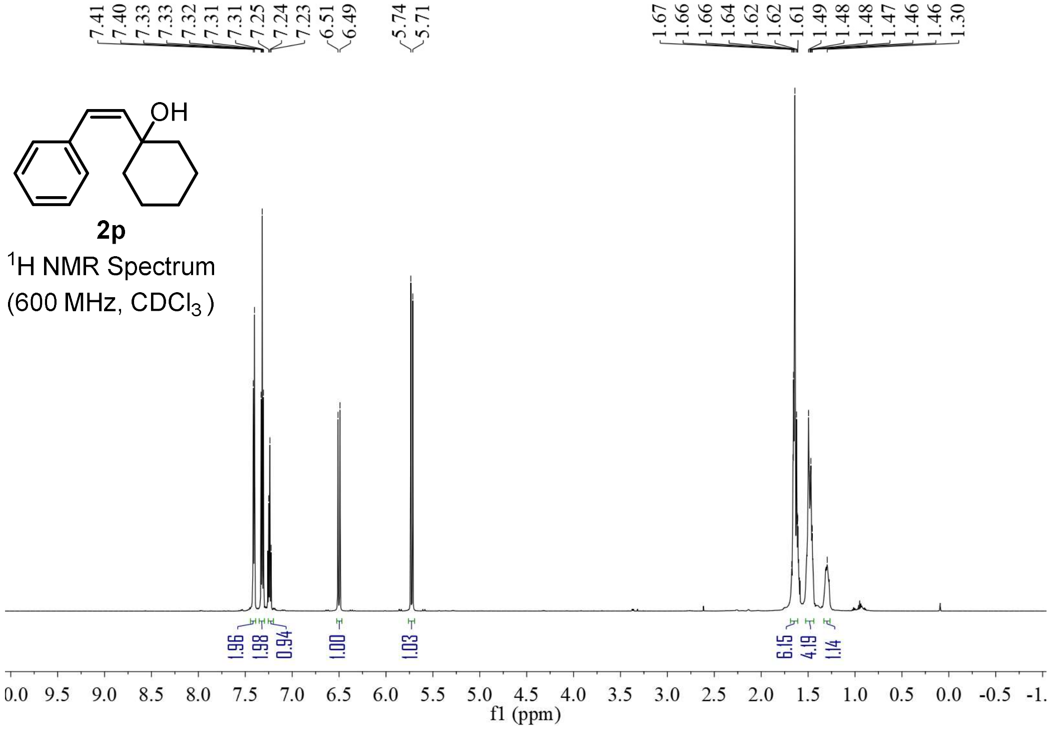
7.41 7.40 7.33 7.33 7.32 7.31 7.31 7.25 7.24 7.23 6.51 6.49 5.74 5.71 1.67 1.66 1.66 1.64 1.62 1.62 1.61 1.49 1.48 1.48 1.47 1.46 1.46 1.30
OH
2p
^{1}H NMR Spectrum
(600 MHz, $CDCl_3$)
1.96 1.98 0.94 1.00 1.03 6.15 4.19 1.14
0.0 9.5 9.0 8.5 8.0 7.5 7.0 6.5 6.0 5.5 5.0 4.5 4.0 3.5 3.0 2.5 2.0 1.5 1.0 0.5 0.0 -0.5 -1.
f1 (ppm)

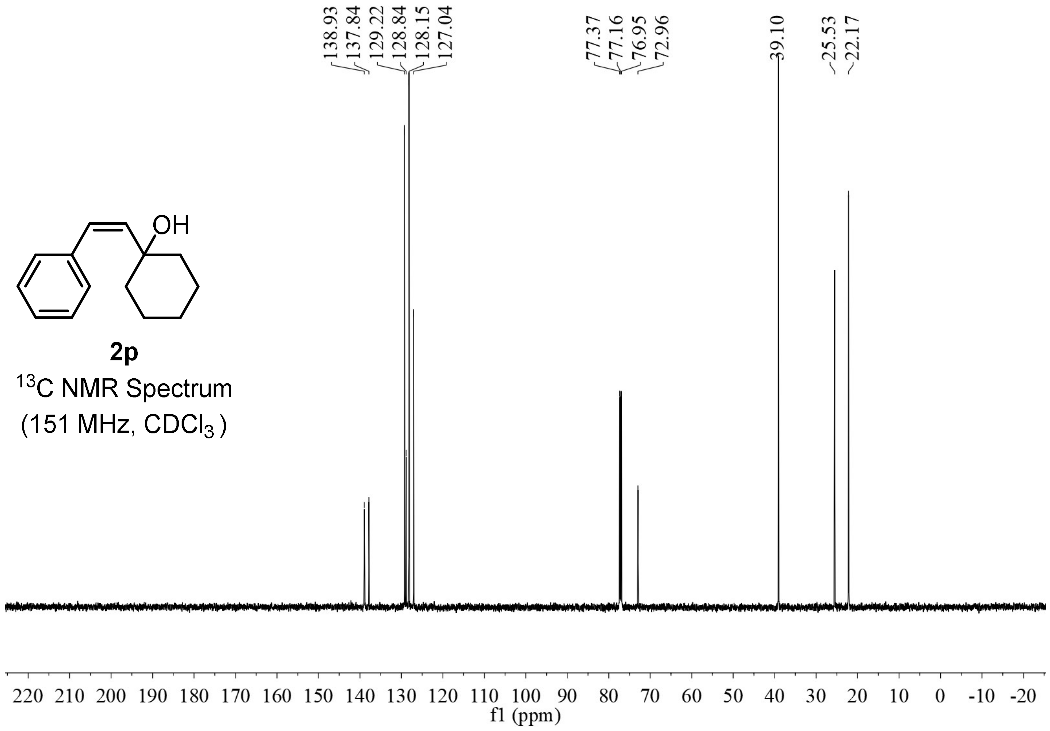
138.93
137.84
129.22
128.84
128.15
127.04
77.37
77.16
76.95
72.96
39.10
25.53
22.17
OH
2p
13C NMR Spectrum
(151 MHz, CDCl3)
220 210 200 190 180 170 160 150 140 130 120 110 100 90 80 70 60 50 40 30 20 10 0 -10 -20
f1 (ppm)

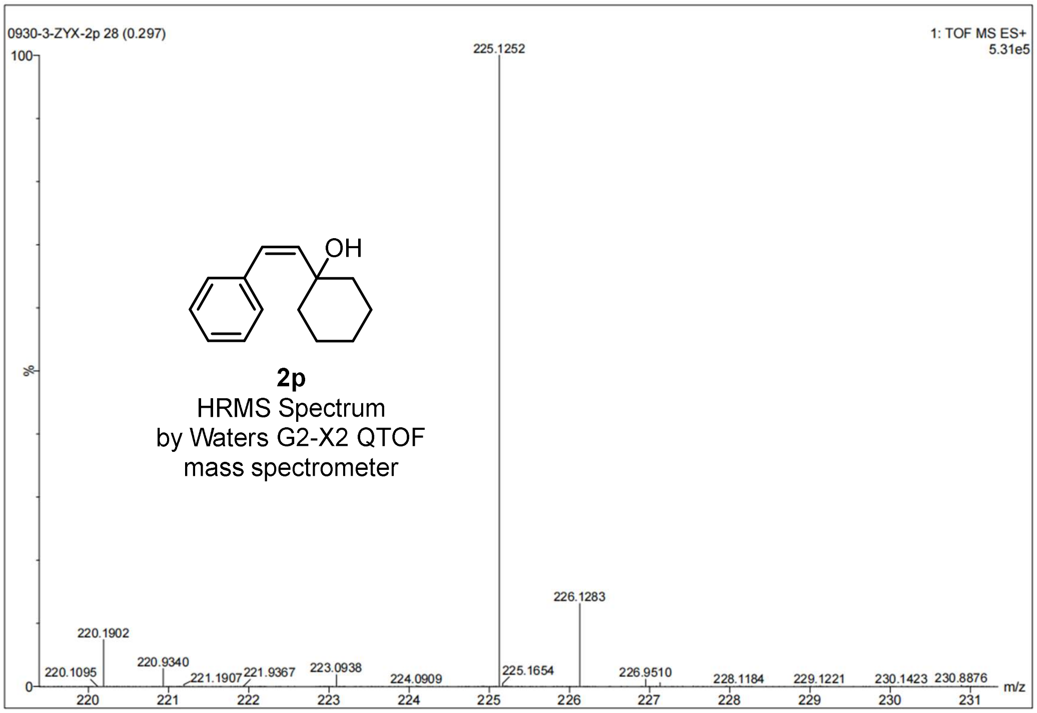
0930-3-ZYX-2p 28 (0.297)
1: TOF MS ES+
5.31e5
100
%
0
225.1252
OH
2p
HRMS Spectrum
by Waters G2-X2 QTOF
mass spectrometer
226.1283
220.1902
220.1095
220.9340
221.1907
221.9367
223.0938
224.0909
225.1654
226.9510
228.1184
229.1221
230.1423
230.8876
m/z
220 221 222 223 224 225 226 227 228 229 230 231

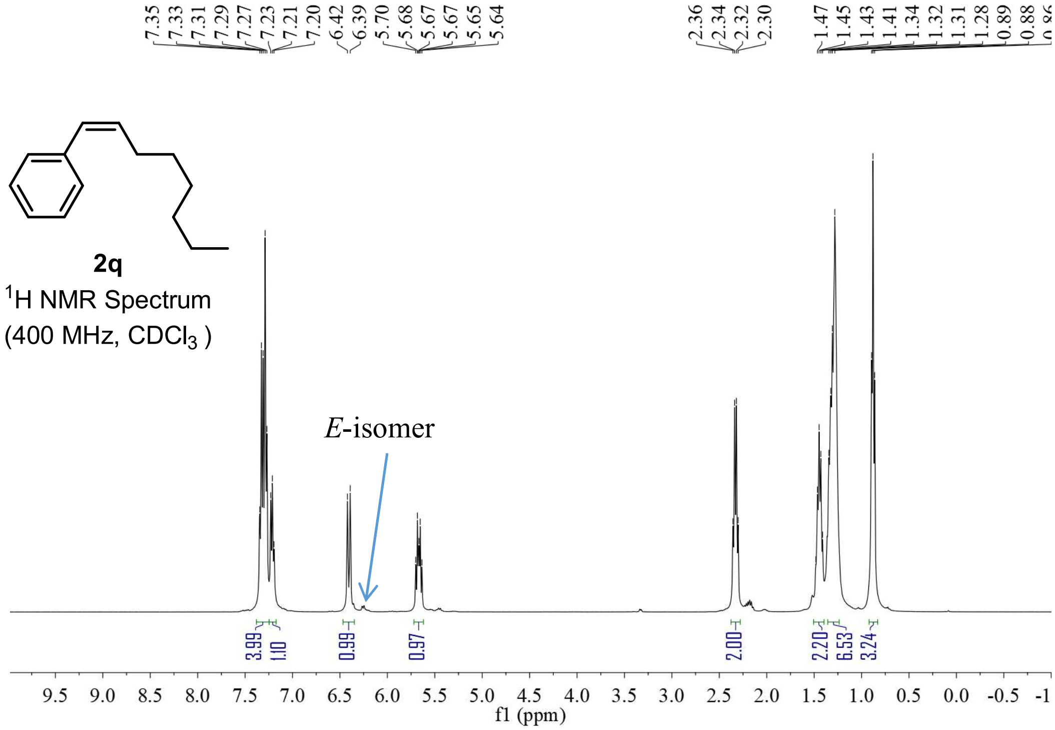
7.35 7.33 7.31 7.29 7.27 7.23 7.21 7.20 6.42 6.39 5.70 5.68 5.67 5.67 5.65 5.64
2.36 2.34 2.32 2.30
1.47 1.45 1.43 1.41 1.34 1.32 1.31 1.28 0.89 0.88
2q
1H NMR Spectrum
(400 MHz, CDCl3)
E-isomer
3.99 1.10 0.99 0.97 2.00 2.20 6.53 3.24
9.5 9.0 8.5 8.0 7.5 7.0 6.5 6.0 5.5 5.0 4.5 4.0 3.5 3.0 2.5 2.0 1.5 1.0 0.5 0.0 -0.5 -1
f1 (ppm)

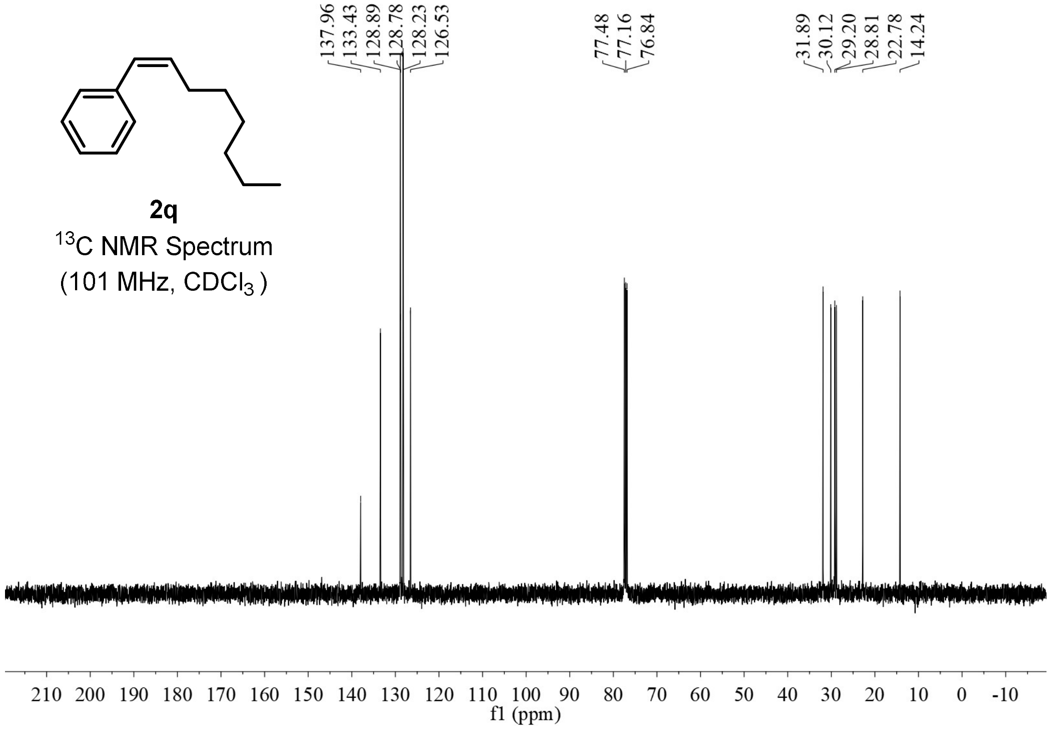
137.96 133.43 128.89 128.78 128.23 126.53
77.48 77.16 76.84
31.89 30.12 29.20 28.81 22.78 14.24
2q
13C NMR Spectrum
(101 MHz, CDCl3)
210 200 190 180 170 160 150 140 130 120 110 100 90 80 70 60 50 40 30 20 10 0 -10
f1 (ppm)

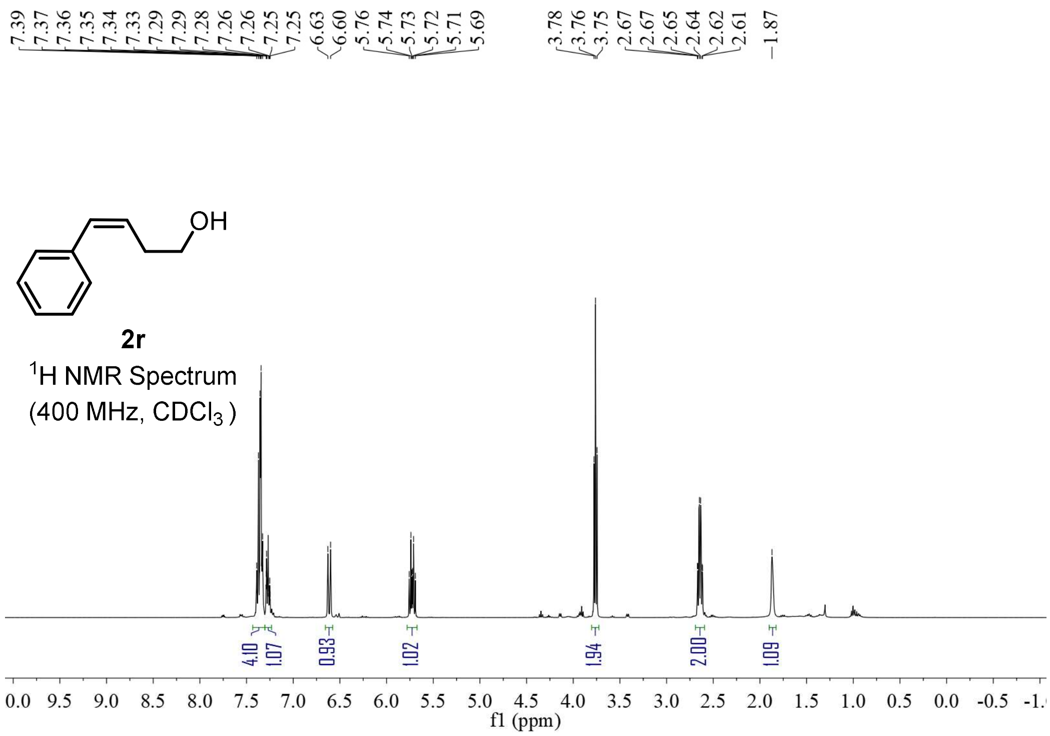
7.39 7.37 7.36 7.35 7.34 7.33 7.29 7.29 7.28 7.26 7.26 7.25 7.25 6.63 6.60 5.76 5.74 5.73 5.72 5.71 5.69 3.78 3.76 3.75 2.67 2.67 2.65 2.64 2.62 2.61 1.87
OH
2r
¹H NMR Spectrum
(400 MHz, CDCl3)
4.10 1.07 0.93 1.02 1.94 2.00 1.09
f1 (ppm)

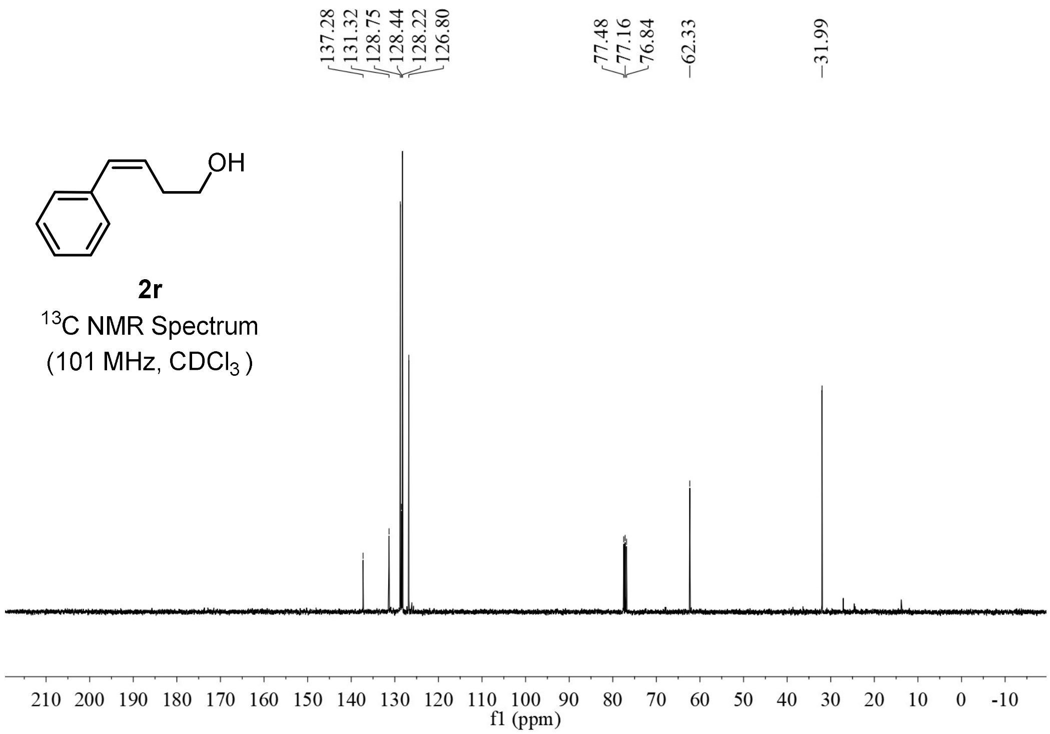
137.28 131.32 128.75 128.44 128.22 126.80 77.48 77.16 76.84 62.33 31.99
OH
2r
¹³C NMR Spectrum
(101 MHz, CDCl3)
f1 (ppm)

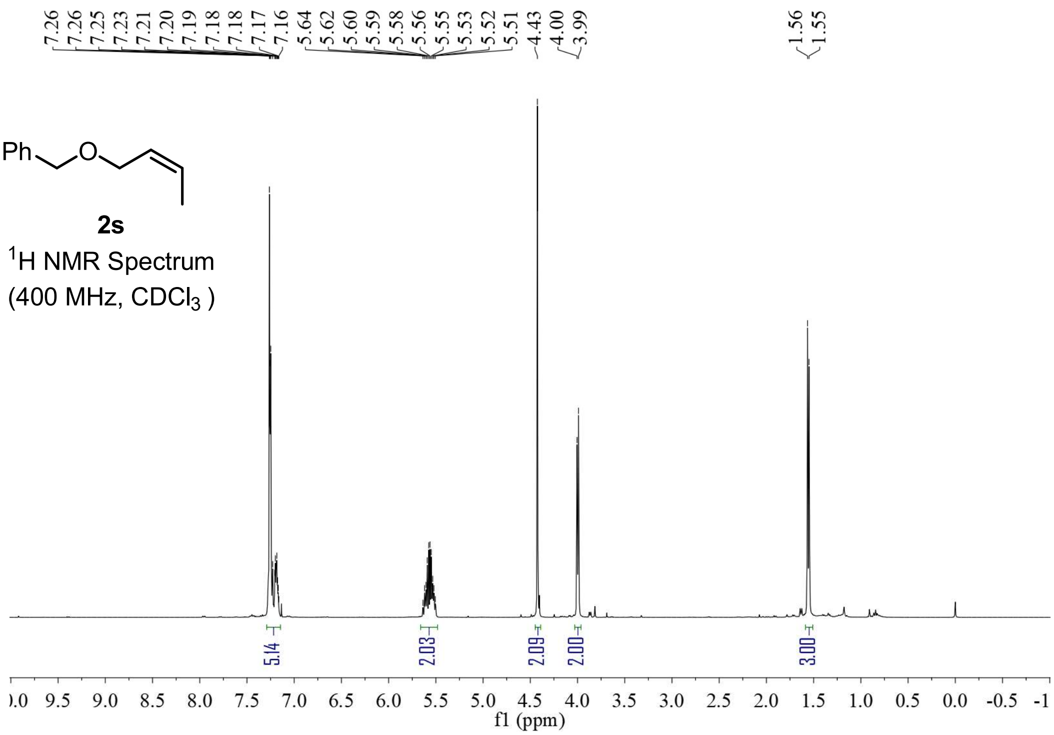

2s

^{1}H NMR Spectrum

(400 MHz, $CDCl_3$)

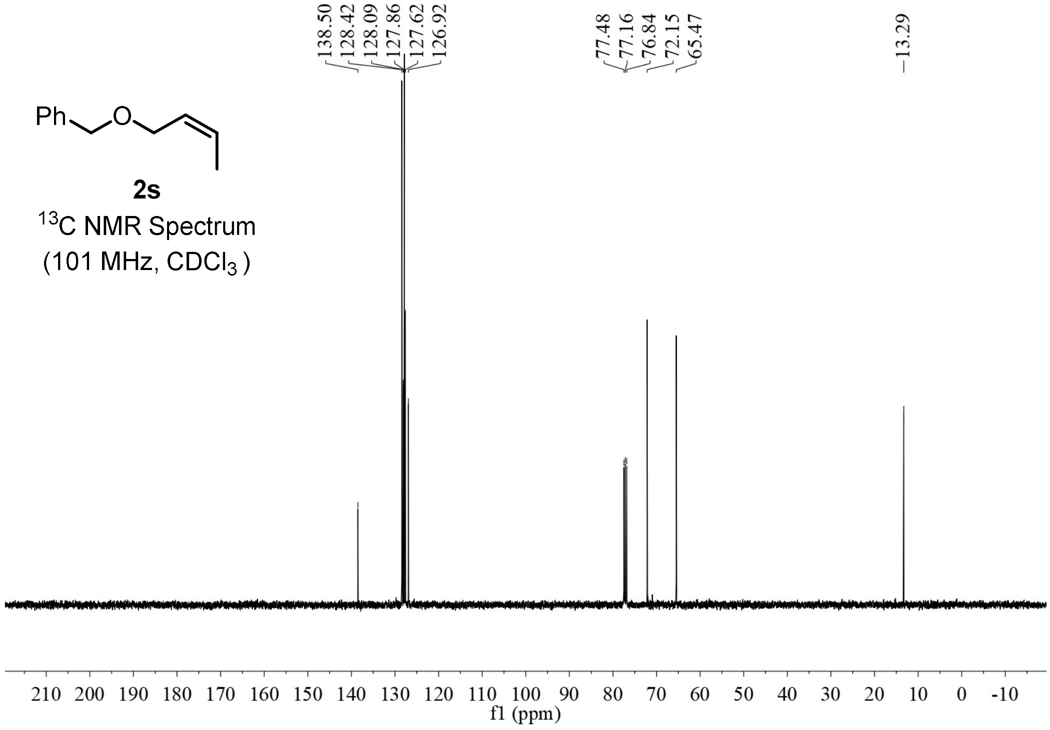

2s

^{13}C NMR Spectrum

(101 MHz, $CDCl_3$)

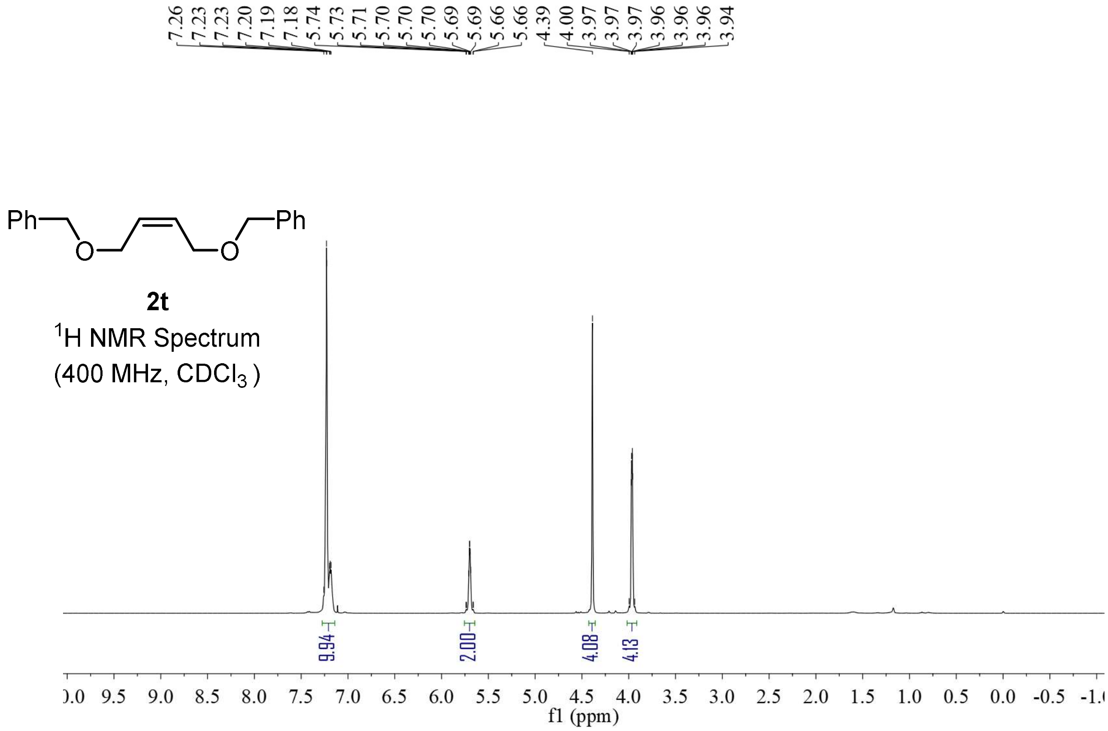
7.26
7.23
7.23
7.20
7.19
7.18
5.74
5.73
5.71
5.70
5.70
5.70
5.69
5.69
5.66
5.66
4.39
4.00
3.97
3.97
3.97
3.96
3.96
3.96
3.94
Ph
O
O
Ph
2t
¹H NMR Spectrum
(400 MHz, CDCl3)
9.94
2.00
4.08
4.13
f1 (ppm)

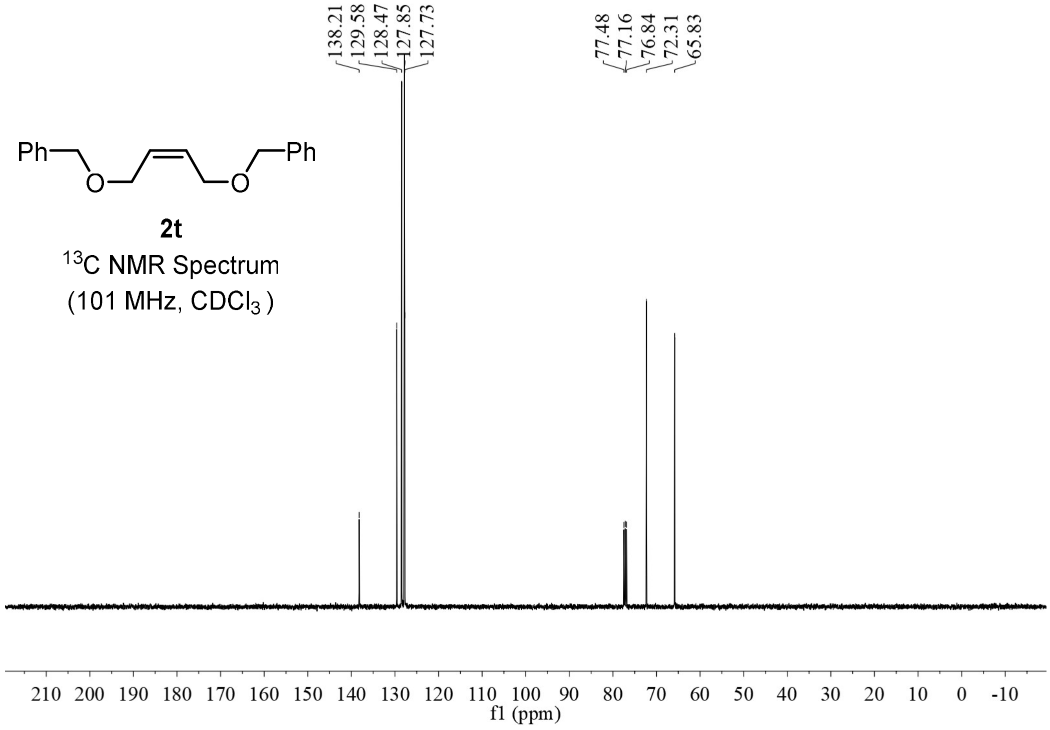
138.21
129.58
128.47
127.85
127.73
77.48
77.16
76.84
72.31
65.83
Ph
O
O
Ph
2t
¹³C NMR Spectrum
(101 MHz, CDCl3)
f1 (ppm)

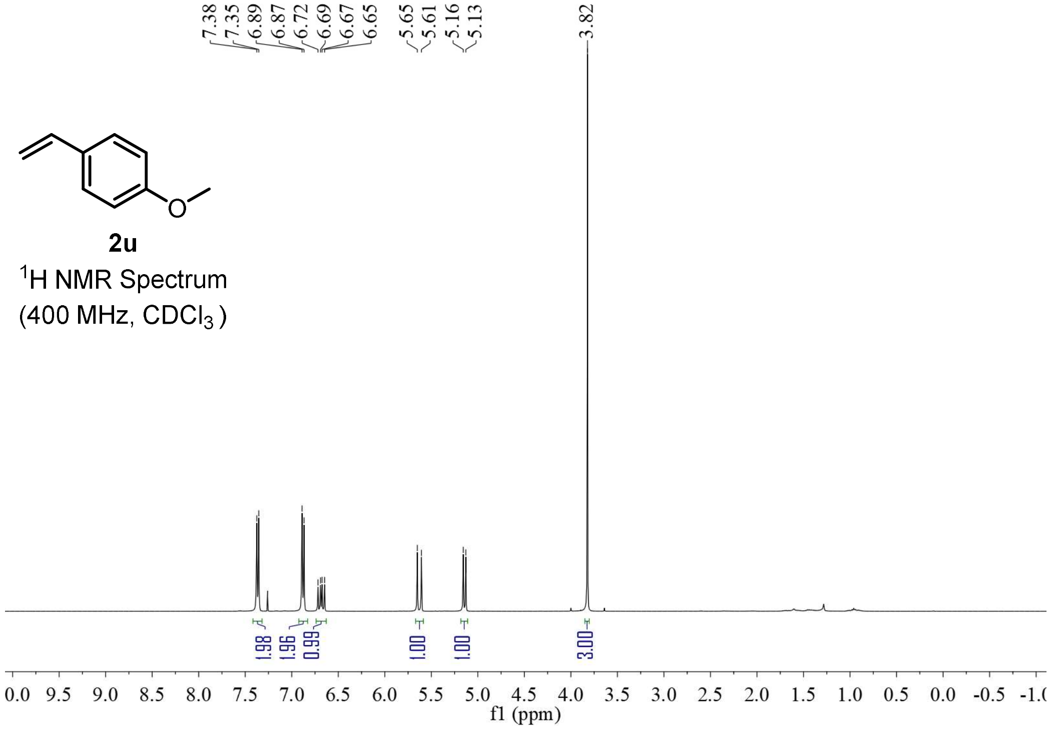
7.38
7.35
6.89
6.87
6.72
6.69
6.67
6.65
5.65
5.61
5.16
5.13
3.82
2u
^{1}H NMR Spectrum
(400 MHz, $CDCl_3$)
1.98
1.96
0.99
1.00
1.00
3.00
f1 (ppm)

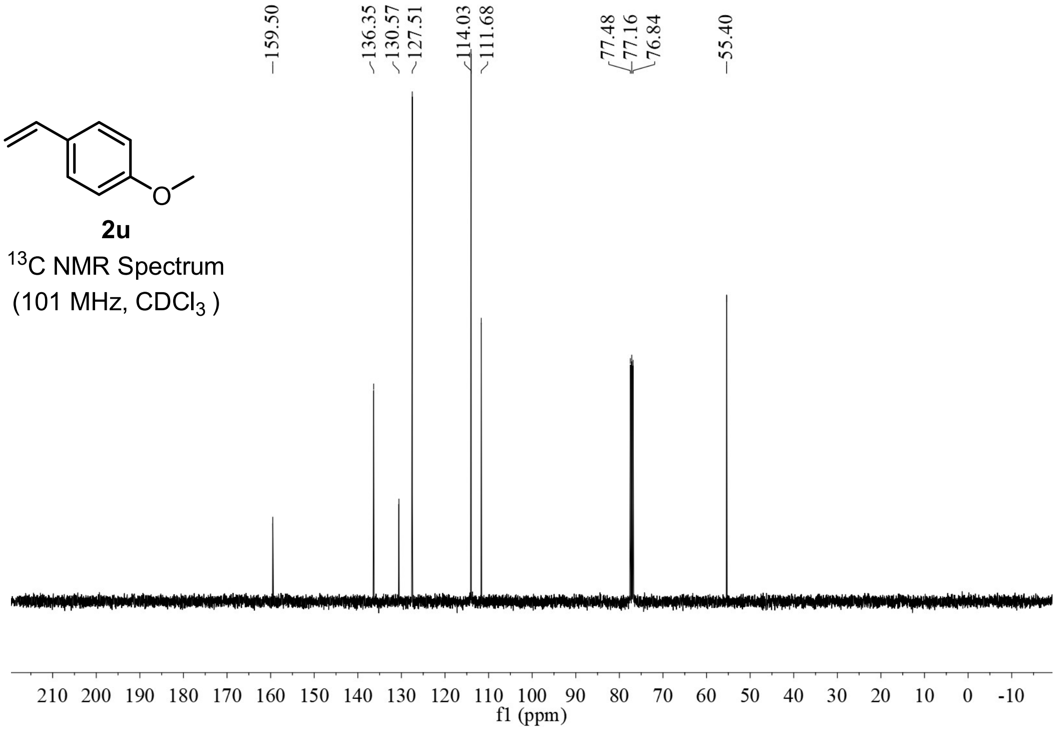
159.50
136.35
130.57
127.51
114.03
111.68
77.48
77.16
76.84
55.40
2u
^{13}C NMR Spectrum
(101 MHz, $CDCl_3$)
f1 (ppm)

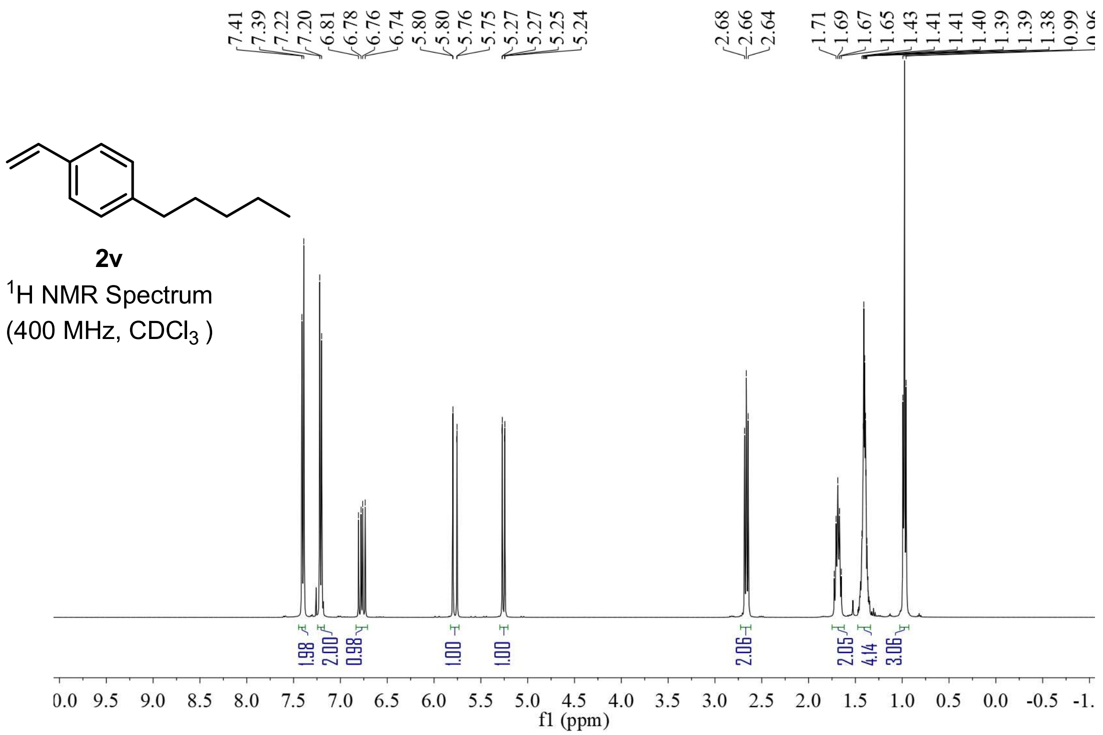
7.41 7.39 7.22 7.20 6.81 6.78 6.76 6.74 5.80 5.80 5.76 5.75 5.27 5.27 5.25 5.24
2.68 2.66 2.64
1.71 1.69 1.67 1.65 1.43 1.41 1.41 1.40 1.39 1.39 1.38 0.99 [illegible]
2v
1H NMR Spectrum
(400 MHz, CDCl3)
1.98 2.00 0.98 1.00 1.00 2.06 2.05 4.14 3.06
0.0 9.5 9.0 8.5 8.0 7.5 7.0 6.5 6.0 5.5 5.0 4.5 4.0 3.5 3.0 2.5 2.0 1.5 1.0 0.5 0.0 -0.5 [illegible]
f1 (ppm)

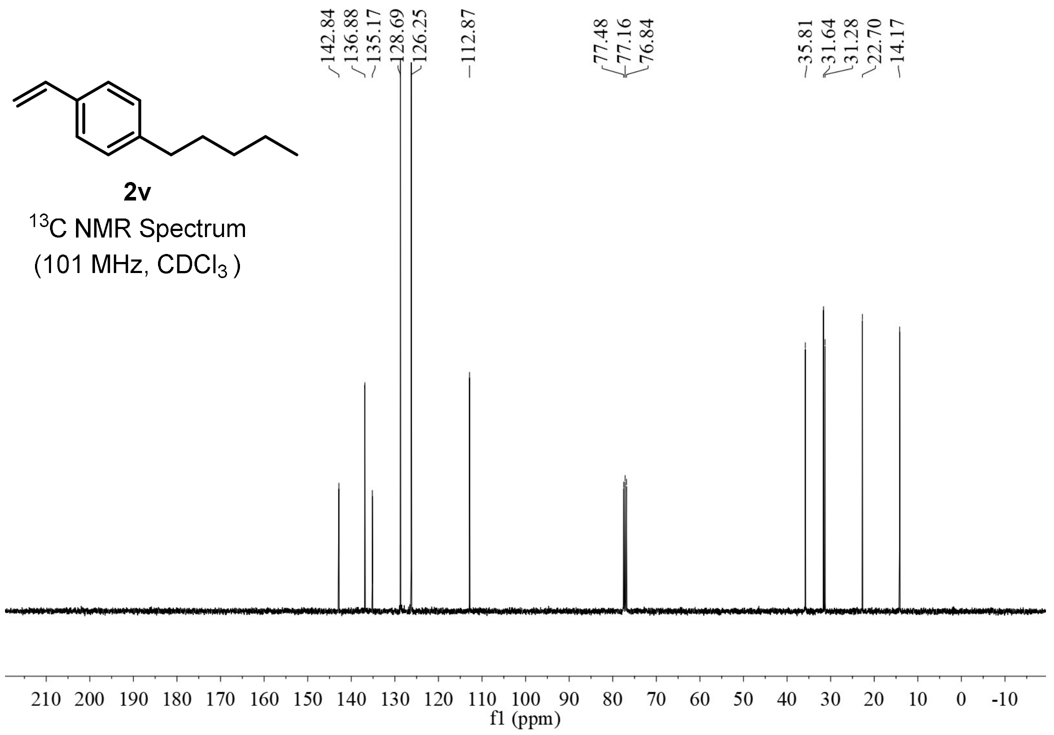
142.84 136.88 135.17 128.69 126.25
112.87
77.48 77.16 76.84
35.81 31.64 31.28 22.70 14.17
2v
13C NMR Spectrum
(101 MHz, CDCl3)
210 200 190 180 170 160 150 140 130 120 110 100 90 80 70 60 50 40 30 20 10 0 -10
f1 (ppm)

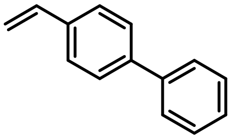

2w
^1H NMR Spectrum
(400 MHz, $CDCl_3$)

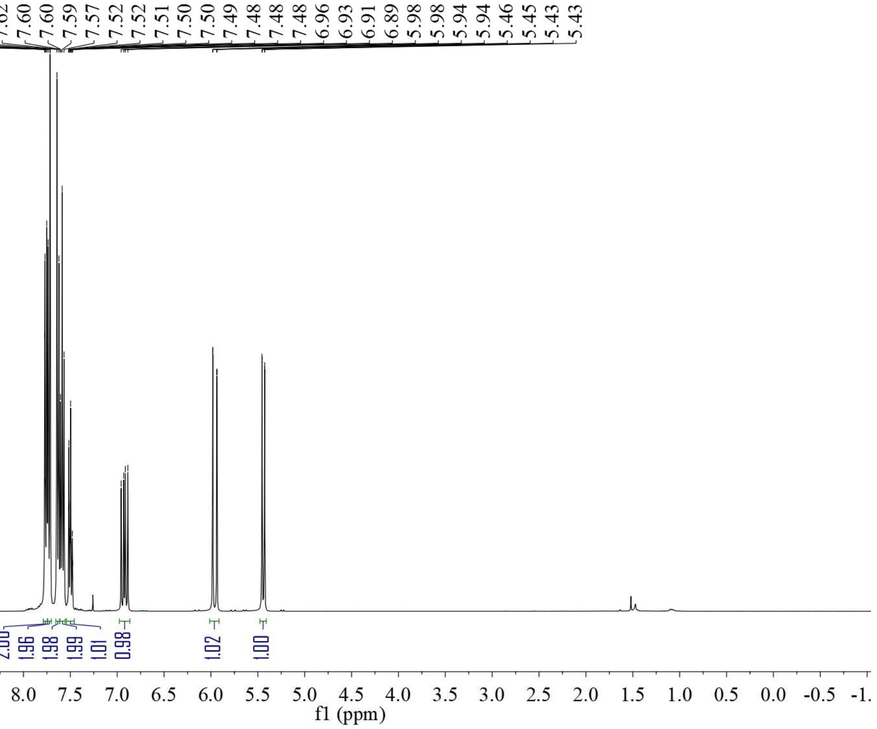

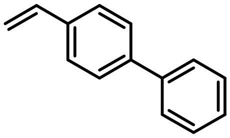

2w
^{13}C NMR Spectrum
(101 MHz, $CDCl_3$)

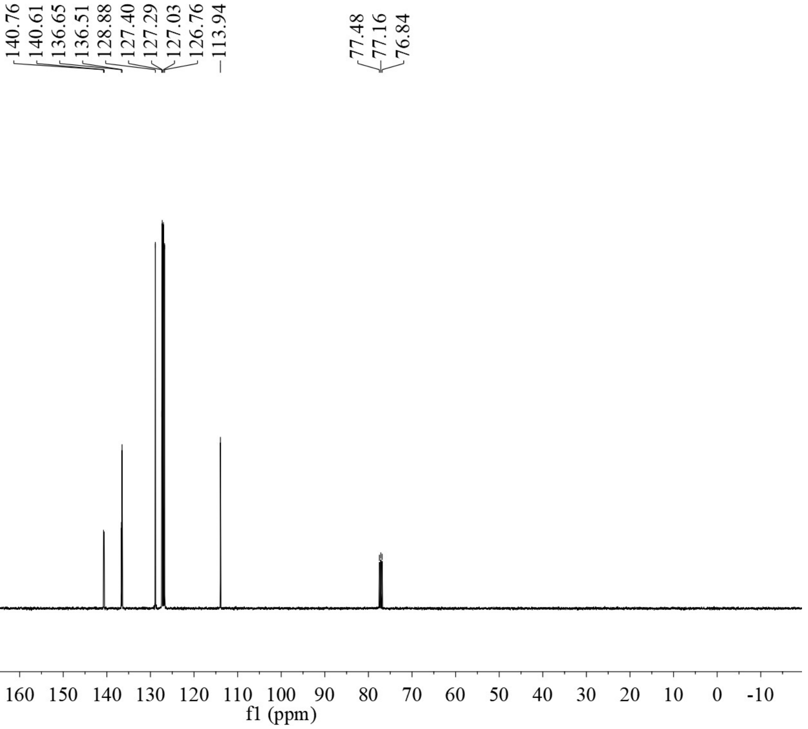

(b) ^{1}H NMR spectrum of 2a-*d*$_2$

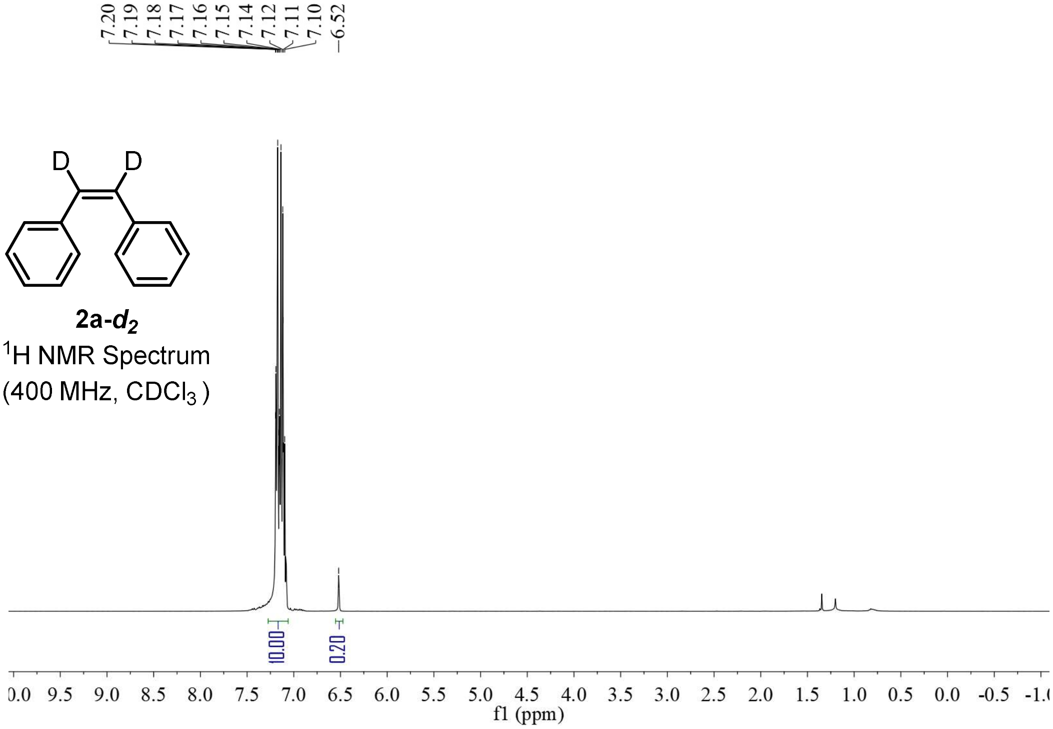

Supplement: Supplementary file 1 [file molecules-27-07213-s001.zip › molecules-1982265-supplementary.pdf]
